# *CEBPD* amplification and overexpression in urothelial carcinoma: a driver of tumor metastasis indicating adverse prognosis

**DOI:** 10.18632/oncotarget.5209

**Published:** 2015-08-17

**Authors:** Yu-Hui Wang, Wen-Jeng Wu, Wei-Jan Wang, Hsuan-Ying Huang, Wei-Ming Li, Bi-Wen Yeh, Ting-Feng Wu, Yow-Ling Shiue, Jim Jinn-Chyuan Sheu, Ju-Ming Wang, Chien-Feng Li

**Affiliations:** ^1^ Department of Pathology, Chi-Mei Medical Center, Tainan, Taiwan; ^2^ Institute of Bioinformatics and Biosignal Transduction, National Cheng Kung University, Tainan, Taiwan; ^3^ Graduate Institute of Medicine, College of Medicine, Kaohsiung Medical University, Kaohsiung, Taiwan; ^4^ Department of Urology, School of Medicine, College of Medicine, Kaohsiung Medical University, Kaohsiung, Taiwan; ^5^ Department of Urology, Kaohsiung Medical University Hospital, Kaohsiung, Taiwan; ^6^ Department of Urology, Kaohsiung Municipal Hsiao-Kang Hospital, Kaohsiung, Taiwan; ^7^ Center for Stem Cell Research, Kaohsiung Medical University, Kaohsiung, Taiwan; ^8^ Department of Pathology, Kaohsiung Chang Gung Memorial Hospital and Chang Gung University College of Medicine, Kaohsiung, Taiwan; ^9^ Department of Biotechnology, Southern Taiwan University of Science and Technology, Tainan, Taiwan; ^10^ Institute of Biomedical Science, National Sun Yat-Sen University, Kaohsiung, Taiwan; ^11^ National Institute of Cancer Research, National Health Research Institutes, Tainan, Taiwan; ^12^ Institute of Clinical Medicine, Kaohsiung Medical University, Kaohsiung, Taiwan; ^13^ Department of Internal Medicine and Cancer Center, Kaohsiung Medical University Hospital, Kaohsiung Medical University, Kaohsiung, Taiwan

**Keywords:** urothelial carcinoma, 8q, CEBPD, amplification, MMP2

## Abstract

The molecular aberrations responsible for the progression of urothelial carcinoma (UC) remain largely obscure. To search candidate driver oncogenes in UC, we performed array-based genomic hybridization (aCGH) on 40 UBUC samples. Amplification of 8q11.21 was preferentially identified in patients who developed disease-specific death (53.8%) and distal metastasis (50.0%) but was barely detected in non-eventful cases (3.7% and 0%, respectively). In order to quantify the expression of candidate genes harbored in 8q11.21, laser-capture microdissection coupled with RT-PCR was performed on 32 of the 40 cases submitted to aCGH. With this, we identified *CEBPD* mRNA expression as most significantly associated with gains of 8q11.21, suggesting amplification-driven expression. By performing *CEBPD*-specific FISH and immunohistochemistry on 295 UBUCs, we confirmed *CEBPD* amplification (21.3%) and overexpression (29.8%) were strongly related to each other (p<0.001). Moreover, both were associated with adverse clinicopathologic features and worse outcomes. Furthermore, the clinical significance of CEBPD expression was also confirmed in an independent cohort comprised of 340 UCs from the upper urinary tract. Interestingly, *CEBPD* knockdown suppressed cell proliferation, migration and, most significantly, cell invasion ability in UC cells. The latter phenotype is attributed to downregulation of MMP2 as identified by RT^2^ Profiler PCR array. Moreover, expression of CEBPD significantly enhanced MMP2 expression and transcriptional activation by directly binding to its promoter region, as confirmed by promoter reporter assay and chromatin immunoprecipitation assay. Conclusively, *CEBPD* amplification is a mechanism driving increased mRNA and protein expression that confers aggressiveness in UC through MMP2-mediated cell invasiveness.

## INTRODUCTION

Urothelial carcinoma (UC), the most common malignancy of the genitourinary tract, originates from the urothelium of the urinary bladder (UBUC) and upper urinary tract (UTUC). Interestingly, UBUC and UTUC share common morphologic, etiologic, and pathological features. Moreover, the gene expression profiles of UCs from different locations are very similar [1]. There is a strong possibility that all UCs, regardless of location, may share a common molecular pathway in carcinogenesis. In past years, investigations focusing on genomic aberrations in UC revealed that complex cytogenetic aberration is characteristic of aggressive behavior. Defining these alterations may help us to understand the genetic hallmarks of tumor progression and help identify new molecular signatures that can be used for better diagnosis, prognostication and the development of more effective therapeutic strategies.

Array-based genomic hybridization is essential in searching for the key chromosomal regions harboring critical genes. In UC, chromosomal gains in 1q, 3p, 6p, 8q, 10p, 17q, and 20q are frequently identified [2, 3]. The gain of chromosome 8q24 harboring *MYC* in particular has been suggested to be associated with UC progression. However, in the literature, the prognostic implications of gains involving different regions of chromosome 8q have been inconsistent, and the derived candidate oncogenes remain largely undefined for UC. To search for candidate oncogenes relevant to tumor progression, we performed aCGH analysis of 40 UBUCs (Table-S1) and identified chromosome 8q as the most significant differentially gained region in UCs (up to 75%) associated with adverse outcomes. Of the whole chromosome 8q, we focused special attention on the gain in 8q11.21, since it was most relevant to the development of distal metastasis and also one of the top-ranking altered regions associated with the development of disease-specific death. Given recurrent gains spanning its DNA locus and significantly increased mRNA expression in UCs with poor outcomes, we specifically selected CCAAT/enhancer binding protein delta (*CEBPD*) at 8q11.21 to evaluate its biological and clinical relevance using cell lines and independent samples.

CCAAT/enhancer binding protein delta (*CEBPD*) is a transcription factor implicated in physiological processes such as cell differentiation, metabolism, inflammation, growth arrest and cell death [4, 5], yet its role in cancer remains much debated. Initially, studies suggested CEBPD acts as a tumor suppressor in leukemia [6-8], prostate cancer [9] and hepatocellular carcinoma [10]. Intriguingly, recent work using a *Cebpd* knockout mouse model to explore mammary tumorigenesis indicated that CEBPD may promote tumor metastasis [11]. One study reported that CEBPD expression level correlates with development of chemotherapy resistance in patients with UC [12]. Based on these seemingly contradictory results, CEBPD could be associated with and contribute to either a better or worse prognosis, depending on the tumor type or cell of origin. To confirm its true function in specific kinds of cancer requires further investigation.

Here we are the first to report that gene amplification is a mechanism that drives CEBPD overexpression in UC, and that its expression correlates with poor clinical prognosis. We confirmed that CEBPD enhances cell growth in UC cell lines by promoting G1-S cell cycle transition. We also showed that CEBPD enhances motility and invasiveness of UC cells via direct promoter binding and active transcription of matrix metalloproteinase-2 (MMP2). These findings reinforce the oncogenic function of CEBPD in UC and contribute to clarifying the molecular mechanisms of how CEBPD promotes tumor metastasis.

## RESULTS

### Recurrent 8q11.21 amplicon spanned *KIAA0146, CEBPD, PRKDC, MCM4,* and *UBE2V2* was preferentially identified in UBUC with poor outcomes

**Figure 1:**
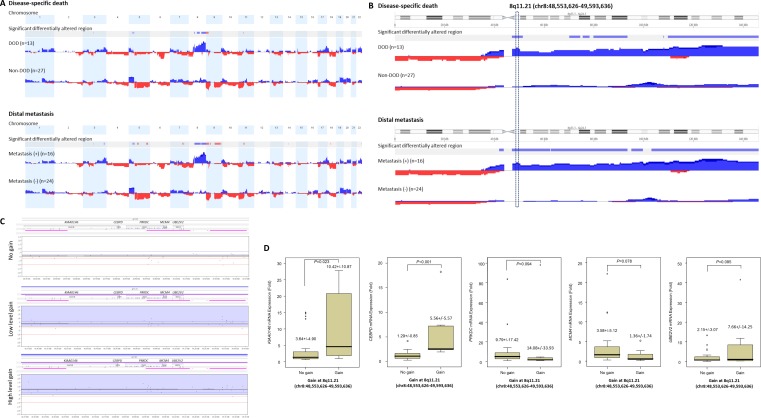
Profiling of genome-wide copy number imbalances in 40 samples with validation By applying Nexus Copy Number™ software, DNA copy number gains (blue) and losses (red) in urothelial carcinoma of urinary bladder (UBUC) are shown in the upward and downward directions, respectively, along the horizontal coordinate of individual chromosomes. Comparing the copy number changes between those who developed disease-specific death (DOD, **A.**, upper panel) and distal metastasis (DM, **A.**, lower panel) to non-eventful cases, the significant differentially altered regions are also illustrated (gains, blue; losses, red). Of note, the most common differentially altered region is 8q. We further finely mapped an amplicon to 8q11.21 (chr8:48,553,626-49,593,636), harboring five named genes in total (*KIAA0146*, *CEBPD*, *PRKDC*, *MCM4*, and *UBE2V2*) which showed most significant preference to those that developed DOD (difference, 50.1%, comparing non-eventful cases, *P*=0.00062) and were also exclusively identified in those with DM (50%, vs. 0% in non-eventful cases, *P*=0.00017) **B**. Representative samples with the various copy number status in 8q11.21 spanning *KIAA0146*, *CEBPD*, *PRKDC*, *MCM4*, and *UBE2V2* are illustrated in the zoom-in view, including no gain (**C.**, upper panel), low level gain (**C.**, middle panel), and high level gain (**C.**, lower panel), respectively. The unit in the vertical axis is the log2 ratio of copy number alterations. Quantitative RT- PCR assay shows that fold expression of *CEBPD* mRNA in the pure UBUC cells from fresh samples is most significantly associated with the presence of genomic gain involving 8q11.21 (*P*<0.01), followed by *KIAA0146* **D.**.

Varying degrees of chromosomal imbalances were detected in all UBUC samples subjected to aCGH profiling. Using Nexus Copy Number™ software, we identified more recurrent regions of gains than deletions across the whole genome in UBUCs. Consistent with the previous literature [13], the most common chromosomal aberrations (Figure-S1) identified in at least half of samples were −9p, +8q, and −5q, which were detected in 60%, 55%, and 50% of the samples, respectively. Other common recurrent alterations with varying extent of involvement included +1q, −2q, −3p, +3q, −4q, +5p, −5q, −6q, +7p, −7q, −8p, −9q, +10p, −10q, −11p, +11q, −13q, −17p, +17q, +18p, −18q, +19q, +20, +22q; we identified these in 20-50% of samples. Computerized by Nexus Copy Number™ software, the recurrent chromosomal aberrations are summarized in Table-S2. Of these, the gains involving 8q showed most significant preference to UBUCs with poor outcomes, exhibiting differential frequencies of 54.4% and 70.8% when comparing patients who developed disease-specific death (dead of disease, DOD) and distal metastasis (DM) to non-eventful cases, suggesting their potential role in tumor aggressiveness (Figure-1A and 1B, Table-S3 and S4). We further finely mapped an amplicon to 8q11.21 (chr8:48,553,626-49,593,636) harboring five named genes in total (*KIAA0146, CEBPD, PRKDC, MCM4*, and *UBE2V2*) which showed most significant preference to those who died of the disease (difference, 50.1%, compared to non-eventful cases, *P*=0.00062). These genes were also exclusively identified in patients with DM (50%, vs. 0% in non-eventful cases, *P*=0.00017) (Table-1, Figure-1B-1C). These findings prompted us to further seek the key driving gene located in this amplification core.

**Table 1:** Five candidate genes located within the core amplified region at 8q11.21 (chr8:48,553,626–49,593,636) detected by aCGH

Gene Symbol	Name	Start	End	Length	Biological Process	Molecular Function
*KIAA0146*	KIAA0146	48336094	48811028	474935		
*CEBPD*	CCAAT/enhancer binding protein (C/EBP), delta	48812028	48813279	1252	regulation of transcription; DNA-dependent, transcription from RNA polymerase II promoter	protein dimerization activity, sequence-specific DNA binding, transcription factor activity
*PRKDC*	protein kinase, DNA-activated, catalytic polypeptide	48848221	49035296	187076	DNA recombination, DNA repair, double-strand break repair via nonhomologous end joining, peptidyl-serine phosphorylation, protein modification process, response to DNA damage stimulus	ATP binding, DNA binding, DNA-dependent protein kinase activity, nucleotide binding, protein binding, transferase activity
*MCM4*	minichromosome maintenance complex component 4	49036046	49052621	16576	DNA replication, DNA replication, DNA replication initiation, DNA unwinding during replication, regulation of transcription	ATP binding, DNA binding, DNA helicase activity, nucleoside-triphosphatase activity, nucleotide binding, protein binding, single-stranded DNA binding
*UBE2V2*	ubiquitin-conjugating enzyme E2 variant 2	49083547	49137007	53461	DNA double-strand break processing, cell proliferation, modification-dependent protein catabolic process, protein polyubiquitination, regulation of DNA repair, regulation of protein metabolic process	protein binding, small conjugating protein ligase activity
*UBE2V2*	ubiquitin-conjugating enzyme E2 variant 2	49083547	49137007	53461	DNA double-strand break processing, cell proliferation, modification-dependent protein catabolic process, protein polyubiquitination, regulation of DNA repair, regulation of protein metabolic process	protein binding, small conjugating protein ligase activity

### *CEBPD* mRNA expression was significantly associated with *CEBPD* gene amplification

To assess the correlation between their expression level and gene amplification status, all of the five genes harbored in 8q11.21 were enrolled for quantifying fold expression of mRNA in LCM-isolated tumor cells from 32 of the 40 fresh samples submitted for aCGH. Real-time RT-PCR revealed that expression levels of *CEBPD* (*P*<0.001) were most significantly upregulated in tumor samples featuring gains on 8q11.21 followed by *KIAA0146* (*P*=0.023) (Figure-1D), thereby reinforcing the role of CEBPD as an amplification-driven gene in UBUC.

### CEBPD expression was significantly associated with gene amplification but not promoter methylation in UBUC; it was also associated with adverse clinical features and worse outcomes

**Figure 2:**
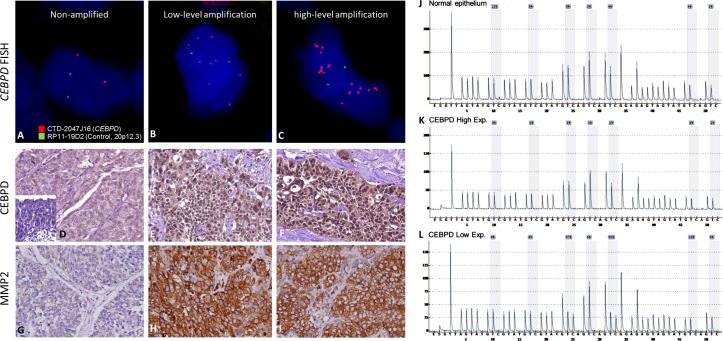
Illustration of *CEBPD* amplification, expression and promoter methylation statuses in UBUC samples with their association to MMP2 expression Representative UBUC samples negative for *CEBPD* amplification **A**. or with low-level **B**. and high-level **C**. amplifications show faint **D**. and significantly increased CEBPD expression (**E.** and **F.**), respectively. Interestingly, the expression of MMP2 is parallel with that of CEBPD in the corresponding cases (**G.**-**I.**). Quantitative pyrosequencing for *CEBPD* promoter CpG islands demonstrated that its methylation levels are usually low, even in those CEBPD low-expressing cases and normal epithelium showing low CEBPD expression (**J.**-**L**.).

Next, we examined the clinical significance of *CEBPD* gene dosage in an independent test set of 295 primary UBUCs. Present in 63 of 295 (21.4%, Table-2 and Figure-2A-2C), *CEBPD* amplification strongly correlated with CEBPD immunohistochemical overexpression (Figure-2D-2F), and both preferentially appeared in cases with more advanced primary tumor (pT) and nodal status (N), as well as in those with higher histological grade, vascular invasion, and higher mitotic activity (Table-2). However, 30 out of 88 CEBPD-overexpressing tumors (34.1%) were not amplified at the *CEBPD* gene locus. These findings indicate that alternative mechanism(s) other than amplification, such as transcriptional regulation, may operate to drive CEBPD overexpression in at least one minor subset of UBUCs (Table-2). Interestingly, in contrast to reports on other cancer types, CEBPD promoter methylation was seldom identified in cancer tissue (10%) and not identified in UBUC cell lines (Figure-2J-2L, Figure-S2, and Table-S5). We also noticed that CEBPD expression is significantly associated with gene amplification (*P*=0.031) but not with promoter methylation (*P*=0.512), suggesting the low frequency of promoter methylation allowed transcriptional activity of the amplified *CEBPD* gene.

**Table 2:** Correlations between *CEBPD* amplification and protein expression and other important clinicopathological parameters in urothelial carcinoma of urinary bladder

Parameter	Category	Case No.	*CEBPD* Gene Amplification			CEBPD Expression		
			Non-Amp.	Amp.	*P*-value	Low	High	*P*-value
Gender^&^	Male	216	169	47	0.780	153	63	0.680
	Female	79	63	16		54	25	
Age (years)^#^		295	65.9+/−12.32	66.6+/−11.90	0.885	65.8+/−12.33	66.7+/−12.00	0.760
Primary tumor (T)^&^	Ta	84	79	5	< 0.001*	78	6	< 0.001*
	T1	88	75	13		67	21	
	T2-T4	123	78	45		62	61	
Nodal metastasis^&^	Negative (N0)	266	217	49	< 0.001*	197	69	< 0.001*
	Positive (N1-N2)	29	15	14		10	19	
Histological grade ^&^	Low grade	56	51	5	0.012*	49	7	0.002*
	High grade	239	181	58		158	81	
Vascular invasion^&^	Absent	246	203	43	< 0.001*	184	62	< 0.001*
	Present	49	29	20		23	26	
Perineural invasion^&^	Absent	275	222	53	0.001*	196	79	0.125
	Present	20	10	10		11	9	
Mitotic rate (mitotic count per 10 high power fields)#		295	13.8+/−14.10	16.8+/−13.66	0.029*	13.2+/−14.32	17.3+/−12.98	0.001*
CEBPD expression^&^	Low Expression	207	202	5	< 0.001*	-	-	**-**
	High Expression	88	30	58		-	-	**-**
MMP2 expression^&^	Low Expression	190	169	21	< 0.001*	163	27	< 0001*
	High Expression	105	63	42		44	61	

& Chi-Square test

# Mann-Whitney U test

* Statistically significant

**Figure 3:**
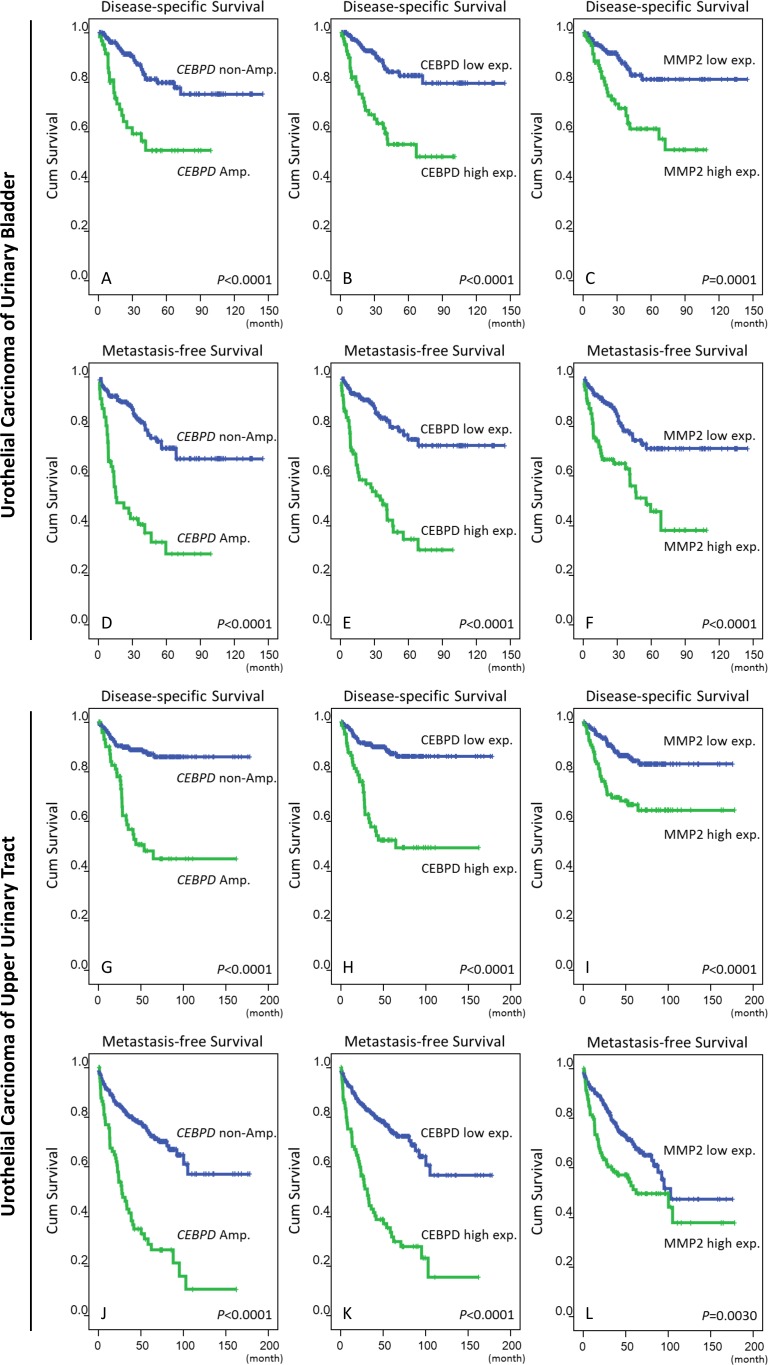
Survival analysis performed by Log-rank test and plotted using Kaplan-Meier methods Both the amplification and high expression of CEBPD are significantly predictive for inferior disease-specific survival (DSS, **A**. and **B**., respectively) and metastasis-free survival (MeFS, **D**. and **E**., respectively) in UBUC. The expression of MMP2, a downstream effector of CEBPD is also a significant predictor for worse DSS **C**. and MeFS **F**. The predicted values of *CEBPD* amplification/expression and MMP2 expression are significant in UC of the upper tract regarding DSS (**G**., **H**., and **I**.) and MeFS (**J**., **K**., and **L**.), respectively.

Clinically, both *CEBPD* amplification and protein expression were strongly predictive of worse outcomes both in terms of disease-specific survival (DSS) and metastasis-free survival (MeFS) (both *P*<0.0001, Figure-3, Table-3). In multivariate comparison, CEBPD expression remained an independently significant prognosticator for disease-specific survival (*P*=0.041) (Table-3), along with pT status, perineurial invasion, and high mitotic activity. In combination with higher pT status it was also predictive of metastasis-free survival (*P*=0.001).

**Table 3:** Univariate log-rank and multivariate analyses for Disease-specific and Metastasis-free Survivals in urinary bladder urothelial carcinoma

Parameter	Category	Case No.	Disease-specific Survival					Metastasis-free Survival				
			Univariate analysis		Multivariate analysis			Univariate analysis		Multivariate analysis		
			No. of event	*P*-value	R.R.	95% C.I.	*P*-value	No. of event	*P*-value	R.R.	95% C.I.	*P*-value
**Gender**	Male	216	41	0.4906	-	**-**	**-**	61	0.2745	-	-	-
	Female	79	11		-	**-**	**-**	16		-	-	-
**Age (years)**	< 65	121	17	0.1315	-	**-**	**-**	32	0.8786	-	-	-
	≥ 65	174	35		-	**-**	**-**	45				
**Primary tumor (T)**	Ta	84	1	< 0.0001*	1	-	< 0.001*	4	< 0.0001*	1	-	0.004*
	T1	88	9		4.566	2.058–10.101		23		3.870	1.125–13.307	
	T2-T4	123	42		24.390	2.702–200.000		50		6.179	1.748–21.846	
**Nodal metastasis**	Negative (N0)	266	41	0.0001*	1	-	0.711	61	< 0.0001*	1	-	0.099
	Positive (N1-N2)	29	11		1.144	0.561–2.335		16		1.662	0.909–3.040	
**Histological grade**	Low grade	56	2	0.0016*	1	-	0.960	5	0.0007*	1	-	0.745
	High grade	239	50		0.960	0.197–4.682		72		1.189	0.419–3.379	
**Vascular invasion**	Absent	246	37	0.0010*	1	-	0.150	54	< 0.0001*	1	-	0.964
	Present	49	15		0.600	0.300–1.203		23		0.986	0.548–1.776	
**Perineural invasion**	Absent	275	44	< 0.0001*	1	-	0.035*	67	0.0003*	1	-	0.152
	Present	20	8		2.476	1.067–5.745		10		1.728	0.817–3.654	
**Mitotic rate (per 10 high power fields)**	< 10	139	12	0.0001*	1	-	0.048*	23	< 0.0002*	1	-	0.065
	> = 10	156	40		1.971	1.006–3.864		54		1.609	0.972–2.665	
**CEBPD Amplification**	Non-amplified	232	30	< 0.0001*	-	-	**-**	42	< 0.0001*	-	-	**-**
	Amplified	63	22		-	-	**-**	35		-	-	**-**
**CEBPD expression**	Low	207	22	< 0.0001*	1	-	0.041*	32	< 0.0001*	1	-	0.001*
	High	88	30		1.912	1.028–3.556		45		2.471	1.459–4.182	
**MMP2 expression**	Low	190	22	0.0001*	1	-	0.616	35	< 0.0001*	1	-	0.492
	High	105	30		1.175	0.625–2.208		42		1.199	0.715–2.010	

* Statistically significant

### Both CEBPD protein overexpression and gene amplification were significantly associated with clinical aggressiveness in UTUC

In order to fully assess the clinical significance of *CEBPD* in the whole spectrum of UCs, we performed further cross-validation for *CEBPD* amplification and expression using an independent UTUC cohort containing 340 cases. We found *CEBPD* amplification and overexpression in 76 (22.4%) and 89 (26.2%) of the UTUC cases, respectively, results similar to those for UBUC (Table-S6). As with UBUC, CEBPD overexpression in UTUC was also significantly associated with gene amplification (*P*<0.001) and only 22.5% of the cases appeared to have CEBPD expression occurring through alternative mechanisms (Table-S6). Of note, both *CEBPD* amplification and expression were also significantly associated with adverse clinicopathologic features, including increments in pT status, nodal metastasis, higher histological grade, vascular and perineurial invasion, and higher mitotic activity (Table-S6). Moreover, both *CEBPD* amplification and expression were strongly predictive of worse DSS and MeFS (all *P*<0.0001). Along with multifocality, nodal metastasis, and perineurial invasion, CEBPD overexpression independently predicted inferior DSS and MeFS in multivariate analysis (Figure-3 and Table-S7).

### CEBPD promotes cell cycle progression, migration, and invasion ability of UC cell lines

**Figure 4:**
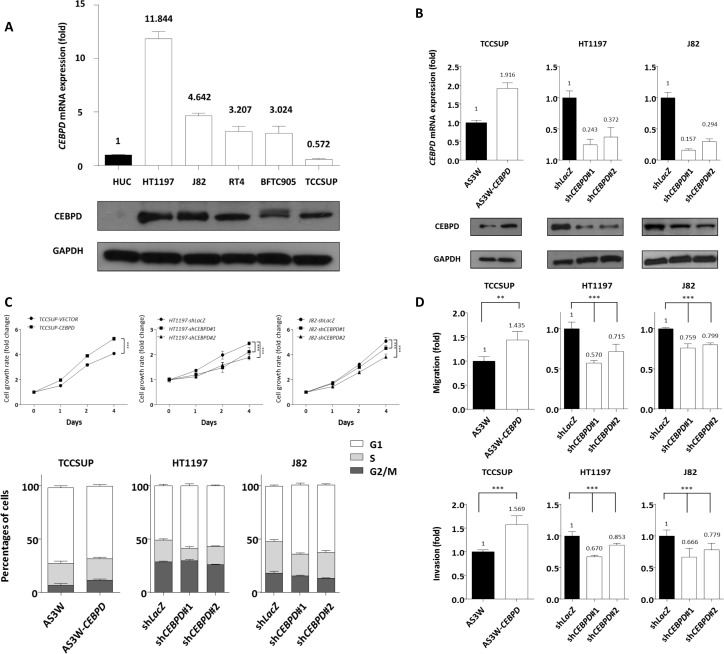
CEBPD expression is associated with tumorigenic potential by enhancing proliferative and metastatic ability of tumor cells Endogenous CEBPD expression levels were determined by quantitative RT-PCR (upper panel) and western blotting assays (lower panel). Compared to non-tumorigenic HUC cell, the majority of UC cells show higher *CEBPD* mRNA and protein expression levels. The expression is even higher in HT1197 which is known to have genomic gain involving the CEBPD gene locus **A**. To further explore the biological functions *in vitro*, stable CEBPD-overexpression and CEBPD-knockdown cell lines have been generated for TCCSUP showing lowest CEBPD endogenous expression and HT1197 and J82 cells, respectively, and the efficiency is confirmed by both quantitative RT-PCR (upper panel) and western blotting assays (lower panel) **B**. Using a 2,3-bis-(2-methoxy-4-nitro-5-sulfophenyl)-2H-tetrazolium-5-carboxanilide (XTT) assay to determine cell viability, we demonstrated positive effects of CEBPD expression on cell proliferation (**C.**, upper panel) in all three cells. Flow cytometric analysis shows the expression of exogenous CEBPD expression promotes G1/S transition and vice versa (**C.**, lower panel). Similar trends are identified for cell migratory (**D.**, upper panel) and invasive ability (**D.**, lower panel). The quantified results are presented as means±sd. Error bars indicate the standard error of the mean. Data represent mean values of three independent experiments. Student's t-test used,*P<0.05, **P<0.01, ***P<0.001).

To investigate the biological effects of CEBPD, we first characterized CEBPD endogenous expression in UC cell lines. As compared with non-tumorigenic urothelial primary cells (HUC) with barely detectable CEBPD expression, the majority of UC cells exhibited elevated CEBPD expression, especially the HT-1197 cell line, which is known to have genomic gain involving the *CEBPD* locus [14] (Figure-4A). We next generated CEBPD overexpressing stable cells in the CEBPD-low expression TCCSUP cell line and performed CEBPD knockdown in CEBPD-high expression HT1197 and J82 cell lines using the stable expression vector or short hairpin RNA (shRNA) for this gene (Figure-4B). To clarify whether CEBPD modulates cell growth, we performed flow cytometric and XTT assays and found that CEBPD promotes G1/S phase transition and enhances cell growth (Figure-4C and Table-S8). Since the expression of CEBPD is notably increased in aggressive UC and correlates with the development of nodal and distal metastasis, we also investigated the involvement of CEBPD in the migration and invasion of UC cells. In a modified Boyden chamber migration assay, expression of exogenous CEBPD increased the migration ability of cells and vice versa (Figure-4D). Similarly, the invasion assay results corroborated those of the migration assay (Figure-4E). These findings demonstrated that CEBPD promotes the migration and invasion of UC cells, prompting us to further explore its mediators and tumor metastasis regulating effects.

### CEBPD enhanced cell invasiveness by transcriptional upregulation of MMP2 expression

**Figure 5:**
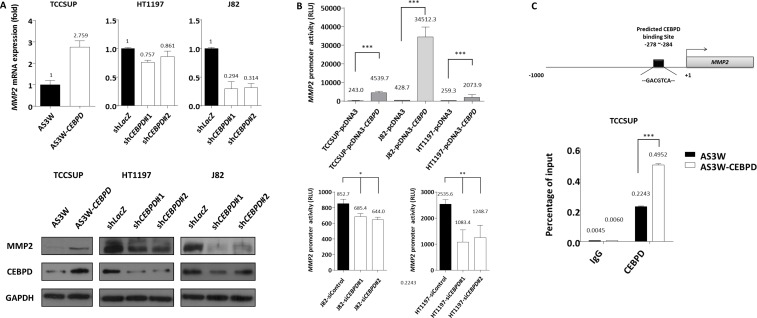
MMP2 is a downstream effector responsible for CEBPD-induced cell invasiveness To validate the results from the Human Tumor Metastasis RT2 Profiler PCR array that identified MMP2 as a potential downstream effector of CEBPD, we first validated MMP2 transcript and protein expression levels and confirmed that exogenous CEBPD expression significantly upregulates MMP2, while its expression is significantly depleted with CEBPD knockdown (**A.**, mRNA, upper panel; protein, lower panel). The reporter vector carrying the *MMP2* promoter was co-transfected with pcDNA3 vector or pcDNA3-*CEBPD* (**B.**, upper panel) either with control or *CEBPD* siRNA (**B.**, lower panel) in UC cells to determine the alteration of promoter activity which disclosed MMP2 transactivity is significantly and positively associated with CEBPD expression. Schematic representation of the promoter region of *MMP2*. The black rectangle indicates predicted CEBPD binding site (**C.**, upper panel). Chromatin from TCCSUP cells with AS3W vector or AS3W-*CEBPD* stable expression are subjected to ChIP assay with a CEBPD antibody and an antibody against immunoglobulin G as negative control. We amplified the precipitated DNA using specific primers targeting the *MMP2* promoter region by quantitative PCR (**C.**, lower panel). The quantified results are presented as means±sd. Error bars indicate the standard error of the mean. Data represent mean values of three independent experiments. Student's t-test used,*P<0.05, **P<0.01, ***P<0.001).

To illuminate potential cellular pathway mediators for cancer invasiveness regulated by CEBPD, we used RT2 Profiler™ PCR arrays to examine the differences in transcript levels of selected signaling molecules involved in metastasis. We selected eight candidate genes according to the selection criteria described in materials and methods (Table-S9). However, considering the statistical significance (*P*-value<0.05) and the consistent trend of alteration of CEBPD expression in three cell lines, we focused on matrix metalloproteinase 2 (MMP2). To further validate this finding we performed qRT-PCR and western blots to assay MMP2 expression levels and found not only MMP2 expression at the mRNA and protein levels were significantly related to CEBPD expression status in all TCCSUP, HT1197, and J82 cells (Figure-5A) but also secreted MMP2 (Figure-S3). Moreover, luciferase activity driven by the *MMP2* promoter was significantly induced by exogenous CEBPD expression in UC cells and vice versa (Figure-5B). Based on these findings we hypothesized that CEBPD activates MMP2 transcription by directly binding to the *MMP2* promoter. By analyzing the *MMP2* promoter nucleotide sequence (−1000/+1), we identified a putative CEBPD binding site located at the *MMP2* promoter. Using quantitative PCR-coupled with chromatin immunoprecipitation (Q-ChIP) assay we further confirmed the binding of CEBPD to the *MMP2* promoter region. The binding affinity was increased with increments of CEBPD expression (Figure-5C). These results suggest that CEBPD enhanced the invasiveness of UC cells through direct binding to the *MMP2* promoter and leads its transcriptional upregulation. We further confirmed MMP2 silencing by shRNA significantly deplete exogenous CEBPD-induced cell migration and invasiveness in TCCSUP cells, confirming the role of MMP2 in CEBPD-driven aggressiveness (Figure-S4).

### MMP2 expression was significantly correlated with CEBPD and associated with adverse clinicopathologic features

Given the significance of MMP2 expression in UC, and the fact that the association between MMP2 and CEBPD expression has not been systematically assessed in UC, we next evaluated its immunohistochemical expression and clinical significance. Interestingly, MMP2 expression was not only positively associated with that of CEBPD (Figure-2G-2I, Table-2, Table-S6) but also with increments of pT and nodal statuses, the presence of vascular invasion and higher mitotic counts (Table-S9). MMP2 expression also conferred more aggressive clinical behavior in both UBUC and UTUC (Table-3 and Table-S7, respectively). These findings further reinforce the role of MMP2 in CEBPD-driving cancer aggressiveness of UC.

## DISCUSSION

UC is a genetically heterogeneous disease with multiple genetic alterations in its development and progression. A comprehensive picture of chromosome imbalances, including severe recurrent abnormalities such as high-level amplifications or homozygous deletions, has been reported for UBUC using cytogenetics and molecular cytogenetic techniques [3]. These studies show that losses at 9q, 9p, 8p, and 11p and gains in 8q, 1q, and 11q are frequently occurring chromosomal aberrations. Chromosome 9 alterations are the most common and are found in more than 50% of UBUCs, regardless of grade and stage, suggesting that its alteration is likely to be an early event, while the evidence suggests that other events, like 8q gains and 8p losses, occur with tumor progression [13, 15, 16].

High-resolution mapping of copy number changes has it possible to identify alterations in many small genomic regions. Some candidate oncogenes and tumor suppressors have been identified among these amplified and deleted loci and have characterized using this approach. For example, the *MTUS1* and *SFRP1* genes on chromosome 8p22 and 8p12-11.1 loci have been identified for their tumor suppression function in UBUC [17, 18]. Regarding chromosome 8q, our results are consistent with those of previous studies which have reported gains involving chromosome 8q as one of the most common events in UBUC. Among the genes harbored in 8q, studies have identified amplification of *MYC* on 8q24 as playing an important role in the development of different human cancers. Alterations of *MYC* gene copy numbers or expression levels are known to occur in UBUC and are associated with late-stage and high-grade tumors [19–21]. Few if any previous reports have attempted to characterize the amplification of other candidate driving oncogenes in the rest regions of 8q. Here we have identified the recurrent amplification core at 8q11.21 that was most relevant to clinical aggressiveness in UBUC, and we have further characterized the *CEBPD* gene as the most significant candidate oncogene featuring amplification-driven expression that is harbored in the chromosome region. We further demonstrated its association with aggressive clinical behavior by cross validation in two independent cohorts of UC. Moreover, we also discovered that CEBPD promotes UC invasiveness via direct binding and transcriptional upregulation of MMP2. Previous reports have shown that CEBPD expression is regulated at the transcriptional, post-transcriptional and post-translational levels. To the best of our knowledge, this report is the first to show its expression can be amplification-driven. Interestingly, while previous research has identified epigenetic silencing of *CEBPD* by promoter hypermethylation in leukemia and breast, liver, and cervical carcinomas in which CEBPD is considered to have tumor suppressive functions [8, 10, 22], in our current work, we noticed a low frequency of *CEBPD* promoter methylation in UBUC samples and cells, suggesting cancer type-specific expression of CEBPD. We also speculate that the lack of promoter methylation allows further transcriptional expression of amplified *CEBPD* genes.

CCAAT/enhancer-binding protein delta (CEBPD) belongs to the CCAAT/enhancer-binding protein family. These proteins function as transcription factors and modulate many biological processes including cell differentiation, motility, growth arrest, proliferation, and death. Deletion of *cebpd* in mice had no overt effect on normal development or the adult life of mice in a pathogen-free environment [23, 24]. Its expression is typically low-to-undetectable, however, a number of extracellular signals can rapidly and transiently induce it. Interestingly, these signals that induce CEBPD often exist in a negative feedback loop [25–27]. The transient nature of CEBPD expression suggests that continued rise of CEBPD levels might lead to adverse outcomes. CEBPD was initially designated as a tumor suppressor based on its physiological functions, including mediating growth arrest and promoting cell death. [6, 9, 10]. However, there is strong evidence that CEBPD may also promote tumor progression in breast cancer. One recent study demonstrated that CEBPD directly inhibits expression of the tumor suppressor FBXW7, resulting in a consequent enhancement of mTOR/AKT/S6K1 signaling, which further suggests CEBPD is necessary for metastatic progression of mammary tumors [11]. Moreover, CEBPD can induce genomic instability through the activation of AURKC expression in response to inflammatory signals in cervical cancer [28]. It also has been proven that increased CEBPD in macrophages promotes nasopharyngeal carcinoma progression by preventing the phagocytosis of tumor cells and inducing immunosuppressive cytokine production [29].

In this study, we demonstrated the oncogenic role of CEBPD in UBUC. We then investigated the molecular mechanisms underlying the metastasis-promoting effects of amplification-driven CEBPD overexpression using mRNA profiling array. With these results, we show that CEBPD regulates expression levels of MMP2 transcript activity in all three UBUC cell lines. We also confirmed positive associations between *CEBPD* amplification and overexpression and MMP2 expression in UC tumor samples.

MMP2 is an enzyme of the matrix metalloproteinase family which is able to degrade components of the extracellular matrix (ECM) and is involved in several physiological and tumorigenic processes, including loss of cell adhesion, ECM remodeling, angiogenesis, cell proliferation, epithelial-to-mesenchymal transition and apoptosis [30]. Elevation of MMP2 has been reported in several cancer tissue types including breast, lung, gastric, ovarian, and bladder cancers. Mostly determined by transcriptional regulation, its elevation endows cancer cells with high invasive and metastatic ability [31]. In UC, the positive association between MMP2 expression and tumor stage and histological grade has been reported based on testing small batches of UBUC without systematic evaluation [32–35]. Moreover, little is known about the molecular regulatory mechanisms of MMP2 expression in UC. Here we demonstrate that CEBPD promotes cell invasiveness by directly regulating MMP2 through transcriptional activation. Our findings suggest that CEBPD plays a critical role in the invasiveness and metastasis of UC in an MMP2-mediated manner. Of note, considerable effort has been put into the investigation of MMP2 over the past decade, not only because of its association with cancer progression, but as an attractive target for pharmacological inhibition [36]. Based on our current findings, we suggest *CEBPD* amplification/overexpression in tumor samples can be a surrogate biomarker to positively select patients that might benefit from MMP2 targeted therapy.

In conclusion, we have identified that *CEBPD* can have an oncogenic role in UC. The dysregulation of CEBPD expression is mainly driven by gene amplification in the lack of promoter methylation in UC. CEBPD expression enhances tumor invasiveness by directly binding to the *MMP2* promoter, resulting in transcriptional upregulation. We also found CEBPD is required for G1/S transition and cell growth. Further investigation is needed to elucidate the molecular mechanisms by which CEBPD exerts it effects on cell proliferation. The findings presented in this study open the possibility that targeting CEBPD and its downstream effectors, such as MMP2, could be a new therapeutic strategy for treating a subset of UC.

## MATERIALS AND METHODS

### Patients and sample collection

To profile the copy number aberrations on a genome-wide scale, an expert pathologist (C.F.L) checked 40 snap frozen UBUC samples with a high percentage of tumor elements (>70%) selected from the BioBank of Chi Mei Medical Center which were then submitted to aCGH. The clinical pathologic features and patient outcomes for the 40 cases submitted to aCGH are summarized in Table-S1. Of these, we used 32 cases with adequate samples for LCM-coupled Real-time RT-PCR to assess the association between candidate gene expression and copy number alterations involving the selected genome region. Afterward we further evaluated the gene which showed the greatest significance, *CEBPD*, by means of FISH and immunohistochemistry in 295 and 340 primary UCUB and UTUC cases, respectively, that were treated consecutively in Chi-Mei Medical Center between 1996 and 2004, as described previously [37, 38]. Twenty UBUCs were randomly selected from the 295 cases for pyrosequencing to further assess the association of CEBPD expression with gene amplification and promoter methylation status. The Institutional Review Board The approved the procurement of clinical samples (IRB10102-004). In these cases, UBUC patients with pT3 or pT4 stage tumors or with nodal involvement received cisplatin-based post-operative adjuvant chemotherapy. However, only 29 of the 106 UTUC patients with pT3 or pT4 stage tumors or with nodal involvement received post-operative adjuvant chemotherapy, since no guidelines supported that protocol during the period from 1996 to 2004. The criteria used for clinicopathological evaluation were essentially identical to those in our previous work [39]. Two expert pathologists (I.W.C and C.F.L) re-evaluated hematoxylin-eosin sections of all cases.

### Performing and analyzing aCGH profiling

The DNA preparation from UBUC samples and aCGH was performed based on our previous methods [40]. In brief, copy number alterations (CNA) were genotyped using 250K single-nucleotide polymorphism (SNP) arrays (Affymetrix) following the suggested protocol. Briefly, genomic DNA was cleaved with Sty1 restriction enzyme and ligated with linkers following amplification by PCR. The PCR products were further purified and digested with DNase I until their sizes ranged from 250 to 1,000 bp. After labeling with biotin, the fragmented products were hybridized to the array, washed in Affymetrix fluidics, then labeled with streptavidin-phycoerythrin conjugates and scanned using a Gene Chip Scanner 3000.

The raw data in CEL format were output into Nexus Copy Number™ software (BioDiscovery) following methods used in our previous work [41, 42]. To finely distinguish the breakpoints in array probes, gains and losses in significant regions of CNAs were defined as log2 ratios of >= +0.20 or =<-0.20, respectively. To identify causal genes exhibiting copy number-driven deregulated expression, we filtered common regions of alteration for consecutive makers where the proportion of analyzed tumor samples was >=20%.

### Laser capture microdissection

As described in our previous work [42], approximately 1500 UBUC cells were isolated from each fresh sample by LCM to quantify the mRNA fold expression of genes of interest.

### RNA extraction and quantitative real-time RT-PCR

Total RNA from cell lines and LCM-isolated tumor cells were extracted using RNeasy Mini Kit (QIAGEN). The isolated RNAs were subjected to RT reactions using SuperScript III (Invitrogen) for cDNA synthesis. Using predesigned TaqMan assay reagents and a StepOne Plus System (Applied Biosystems), we measured mRNA abundance. Pre-designed TaqMan assay reagents are detailed as follows: *KIAA0146*, Hs00218603_m1; *CEBPD*, Hs00270931_s1; *PRKDC*, Hs00179161_m1; *MCM4*, Hs00907398_m1; *UBE2V2*, Hs00163342_m1; *MMP2*, Hs01548727_m1; and *POLR2A* (a.k.a, RNA polymerase polypeptide A), Hs01108291_m1. We calculated the fold expression of target genes relative to normal adjacent tissues or vehicle controls by comparative Ct method after normalization to *POLR2A* as the internal control.

### Fluorescence in situ hybridization

We assessed the *CEBPD* gene copy number in cancer tissue samples on FFPE sections by locus-specific FISH. A laboratory-developed bacterial artificial chromosome (BAC) probe (CTD-2047J16) spanning *CEBPD* at 8q11.21 was labeled with spectrum orange. According to our current genomic profiling data, there were no CNA at 20p12.3, and a BAC probe targeting this region (RP11-19D2) was selected as the reference probe and labeled with spectrum green. We determined the average numbers of orange and green signals by examining approximately 200 tumor cells for each specimen. Gene amplification was defined as a ratio of the gene probe signal to the control probe signal exceeding two.

### Immunohistochemistry, interpretation and scoring

The immunohistochemical methods for FFPE sections have been previously described and modified with primary antibodies targeting CEBPD ( 1;200, ab65081, Abcam ) and MMP2 ( 1:100, ab31057, Abcam ) [43, 44]. A labeling index was recorded as 0~4% (0+), 5~24% (1+), 25~49% (2+), 50~74% (3+) and 75~100% (4+) of tumor cells that displayed strong nuclear staining. For both CEBPD and MMP2, Only cases with 3+ and 4+ immunoexpression were regarded as high expression and those with 0+ to 2+ as low expression.

### Evaluation of *CEBPD* gene promoter methylation using pyrosequencing

Promoter methylation of *CEBPD* was quantified by pyrosequencing. For this purpose, PCR amplification and sequencing primers were designed by PyroMark Assay Design Software 2.0. Primer sequences were *CEBPD*-forward:

GAAGGTTTTGGAGTGTTGGTAGA and *CEBPD*-reverse: biotin-CCCCCTCTCAATTCCTCC, with the amplicon in *CEBPD* promoter region being 159 bp (from 47,738,318 to 47,738,476 on chromosome 8q11.2, based on NCBI database). Bisulfate treatment and cleanup of DNA was performed by using EpiTect 96 Bisulfite Kit (Qiagen). PCR amplification of target region was performed by using PyroMark PCR Kit (Qiagen). The biotin-labeled PCR product was captured by Streptavidin-Sepharose HP (Amersham Pharmacia). PCR products bound on the beads were purified and made single stranded using a Pyrosequencing Vacuum Prep Tool. The sequencing primers, *CEBPD*-S1: GGTAGAGGGAGTGTTAT and *CEBPD*-S2: GAGTAGTAGAGAGGGTATTTTTTTG, were annealed to the single-stranded PCR product. We used the PyroMark Q24 system (Qiagen) for pyrosequencing and the PyroMark Q24 Software for quantitation of cytosine methylation. Methylation is called when the average methylation percentage of all CpG islands tested was higher than 19.324%, which was the mean+3 s.d. of the average methylation percentage of CpG islands of normal epithelia, as described previously [45].

### Cell culture and establishment of stable CEBPD expression cells

RT4, BFTC905, BFTC909, TCCSUP cell lines were purchased from the Food Industry Research and Development Institute of Taiwan. Cell culture condition was performed as previously described [46]. HT1197, J82, were purchased from ATCC (Manassas, VA 20108, USA). We cultured J82 in Dulbeco's modified Eagle's medium containing 10% FBS, 1% Penicillin-Streptomycin, 100X (GIBCO). HT1197 cells were cultured in Eagle's Minimum Essential Medium containing 10% FBS, 1% MEM Non-Essential Amino Acids Solution, and 1% Antibiotic-Antimycotic, 100X(GIBCO). A non-tumoral urothelial primary cell, HUC (ScienCell Research Laboratories, San Diego, CA), was used as a control and maintained using the suggested medium and conditions. To generate stable CEBPD expression cell lines. TCCSUP cells were plated in 6-well plate at a density of 1×10^6^ per well, and then were infected by lentivirus for 48 hours. We purchased the pLKO-AS3w-puro expression vector from the National RNAi Core Facility (Academia Sinica, Taipei, Taiwan). Lentiviral vector express CEBPD (pLKO-AS3w-CEBPD-eGFP) was constructed according to protocol. The DNA sequence containing CEBPD's open-reading frame (ORF) was cloned from Hela cells using specific primers and inserted into the pLKO-AS3w-puro vector. The pLKO-AS3w-eGFP-puro plasmid was used as a negative control. Virus was produced as previously described [47].

### Lentiviral short hairpin RNA in knockdown

We purchased the lentiviral expression plasmids from the National RNAi Core Facility located at the Genomic Research Center of the Institute of Molecular Biology, Academia Sinica, Taiwan. Virus was produced as previously described [47]. Viral supernatants were harvested in the conditioned medium. After determining the viral infection efficiency, we used these viral supernatants to infect cells for 48 hours. The shRNA sequences in the lentiviral expression vectors were: shLacZ, 5′-CCGGTGTTCGCATTATCCGAACCATCTCGA GATGGTTCGGATAATGCGAACATTTTTG-3, sh*CEBPD*#1, 5′-CCGGGCCGACCTCTTCAACAGCAATCTCG AGATTGCTGTTGAAGAGGTCGGCTTTTT-3, sh*CEBPD*#2, 5′-CCGGGCCGACCTCTTCAACAGCAATCTCG AGATTGCTGTTGAAGAGGTCGGCTTTTT-3, shVoid, 5′-CCGGAGTTCAGTTACGATATCATGTCTCGA GACATTCGCGAGTAACTGAACTTTTTT-3′, sh*MMP2*#1, 5′-CCGGGCAGACATCATGATCAACTTTCTCG AGAAAGTTGATCATGATGTCTGCTTTTTG-3′, sh*MMP2*#2, 5′-CCGGCCAAAGTCTGAAGAGCGTGAACTCG AGTTCACGCTCTTCAGACTTTGGTTTTTG-3′.

### Western blot assays

Equal amounts of protein extract were separated by 4%–12% gradient NuPAGE gel (Invitrogen), and then the protein was transferred onto polyvinylidene difluoride (PVDF) membranes (Amersham) and blocked with 5% skimmed milk in TBS-0.1% Tween20 buffer at room temperature for one hour. Afterwards, the membranes were probed with antibodies against proteins of interest at 4°C overnight and then incubated with the secondary antibody at room temperature for one hour. We used enhanced chemiluminescence reagents (Amersham, Piscataway, NJ) to visualize the targeted proteins, which were then quantitated by densitometry. Primary antibodies used were as followings: CEBPD (1:250, SC-636, Santa Cruz), MMP2 (1:1000, Abcam) and glyceraldehyde-3-phosphate dehydrogenase (GAPDH) (1:10000, Chemicon).

### Cell cycle analysis with flow cytometry

We harvested 80% of confluent cells in 6cm dishes and rinsed them in HBSS, then fixed them in ice-cold 70% ethanol, and stored them at −20°C. Before flow cytometry analysis, cells were pelleted and re-suspended in PI/RNase Staining Buffer (BD Biosciences). We stained cells in the dark for 15 minutes. During analysis ten thousand events were acquired and the proportions of cells in each cycle phase were calculated using software. We performed each experiment at least three times.

### XTT (2,3-Bis-(2-Methoxy-4-Nitro-5-Sulfophenyl)-2H-Tetrazolium-5-Carboxanilide) assay

Cell viability was determined by using XTT (Sigma) according to the product manual. Briefly, cells were plated in 96-well plates at their appropriate densities (3,000~5,000cells/well). We then incubated the cells at 37°C in a humidified atmosphere containing 5% CO2. After 24, 48, 72 hours of incubation, the culture medium was removed and an XTT reaction mixture was added to each well, then incubated for four hours at 37°C. The absorbance was measured at a wavelength of 450 nm against a reference wavelength of 630 nm in a microplate reader.

### Migration and invasion assays

Migration and invasion ability of cells were determined by Boyden chamber technique (transwell analysis). The cell migration assay was carried out with Falcon HTS FluoroBlok 24-well inserts (BD Biosciences). The cell invasion assay was performed using the 24-well Collagen-Based Cell Invasion Assay (Millipore). Briefly, we rehydrated each insert by adding serum-free medium, then replaced it with serum-free suspension with equal amounts of cells in the upper chamber, and incubated them for 12 to 24 hours to let cells migrate toward/invade the lower chamber containing 10% FBS. After removing the non-invading cells in the upper chamber, cells invading through the inserts were stained with provided dye, dissolved in extraction buffer, and transferred to 96-well plates for colorimetric reading at 560 nm.

### Real-time PCR gene array

To explore potential mediators of CEBPD implying its pro-metastatic function, we performed RNA expression profiles. RNA was extracted from TCCSUP, J82, HT1197 control and CEBPD overexpression/knockdown comparator cell lines. Subsequently, total RNA was reverse transcribed to cDNA using methods mentioned before. We then performed real-time PCR on each sample using the Human Tumor Metastasis RT2 Profiler PCR array (PAHS-028, SABioscience) according to the manufacturer's instructions. Expression was normalized to housekeeping genes and presented as fold expression relative to the corresponding controls. Those genes differentially expressed in at least two of three cell lines with >1.75-fold upregulation or downregulation between CEBPD-overexpressing or CEBPD-knockdown and control were enrolled for further selection.

### Enzyme-Linked Immunosorbent Assay (ELISA)

Human MMP2 ELISA kit was purchased from abcam (Cambridge, MA) and test was formed under manufacturer's instruction. Briefly, 5×10^6^ cells were seeded on 100 mm dishes. The cells were incubated with 8ml serum-free culture medium for 48hrs. The supernatants were then collected and the levels of MMP2 were determined by the absorbance value at 450 nm (GloMax®-Multi Detection System, Promega).

### Small interfering RNA (siRNA)

For siRNA studies, the cells were transfected with pre-designed, validated, human si*CEBPD* RNA or negative control siControl RNA (Ambion, Inc., Austin, TX) in the presence of an Lipofectamine® RNAiMAX reagent (Invitrogen, Carlsbad, CA), as per the manufacturer's instructions.

### Luciferase reporter assays

We constructed the pcDNA3-CEBPD plasmid according to methods previously described [48]. The promoter fragments of MMP2 ( −992 to −191) were cloned using polymerase chain reaction (PCR) of genomic DNA from HeLa cells and inserted into the promoterless pGL3-Basic vector (Promega, Madison, WI). MMP2 reporter plasmids and pcDNA3-CEBPD plasmids or their corresponding control empty pcDNA3 vector plasmids were co-transfected into cells using PolyJetTM (SignaGen Laboratories, Gaithersburg, MD) transfection reagent according to the manufacturer's instructions. After transfection for 48 h, cells were prepared and subjected to One-Glo Luciferase Reporter Assay (Promega) according to the manufacturer's instructions. Luciferase activity was normalized to cell number.

### Chromatin immunoprecipitation

We performed the chromatin immunoprecipitation (ChIP) assay using Magna ChIP A/G Chromatin Immunoprecipitation Kit (Millipore Cat no.: 17–10085; Temecula, CA, USA). Briefly, cells were fixed with 1% formaldehyde for 15 min. The cross-linked chromatin was then prepared and sonicated to an average size of 500–1000 bp. DNA fragments were immunoprecipitated at 4°C overnight with antibodies that were specific to CEBPD or control immunoglobulin G. The cross-linking was reversed and purified, the specific DNA fragments were quantitated by qRT–PCR and normalized to input. The qRT–PCR primers for MMP2 are same as described before [49].

### Statistical analyses

Statistical analyses were done using the SPSS 10 software package (SPSS Inc., IL. Chicago). We assessed associations among different variables and *CEBPD* amplification/expression and MMP2 expression using the, Mann-Whitney U test, or Chi-square method, as appropriate. The follow-up duration ranged from 1 to 109 months (median, 23) for UBUC and 1 to 176 months (median, 38) for UTUC, respectively. For survival analyses, we plotted the disease-specific survival (DSS) and metastasis-free survival (MeFS) using Kaplan-Meier curves and computed the prognostic differences by log-rank test at the univariate level. All variables that were significant at the univariate level were entered into a Cox multivariate regression model except for *CEBPD* gene amplification, which appeared to be strongly associated and showing overlapping clinicopathologic and biological significance with CEBPD expression. For all analyses, two-sided tests of significance were used with P < 0.05 considered significant.

## SUPPLEMENTARY MATERIAL FIGURES AND TABLES



## Abbreviations

UC: Urothelial carcinoma
aCGH: array-based comparative genomic hybridization
CEBPD: CCAAT/enhancer binding protein delta
MMP2: matrix metalloproteinase-2
DSD: disease-specific death
DM: distal metastasis
DSS: disease-specific survival
LCM: laser capture microdissection
qRT-PCR: Quantitative reverse transcription polymerase chain reaction
FISH: fluorescent in situ hybridization
HUC: Urothelial primary cell
shCEBPD: short-hairpin RNAs against CEBPD expression
Q-ChIP: quantitative PCR-coupled with chromatin immunoprecipitation
