# Supplementary material for: CEBPD amplification and overexpression in urothelial carcinoma: a driver of tumor metastasis indicating adverse prognosis
Source: Oncotarget. 2015 Aug 17;6(31):31069–84. doi: 10.18632/oncotarget.5209 (PMC4741589; doi:10.18632/oncotarget.5209)
Supplement: Supplementary file 1 [file oncotarget-06-31069-s001.pdf]

CEBPD amplification and overexpression in urothelial carcinoma: a driver of tumor metastasis indicating adverse prognosis

Supplementary Material

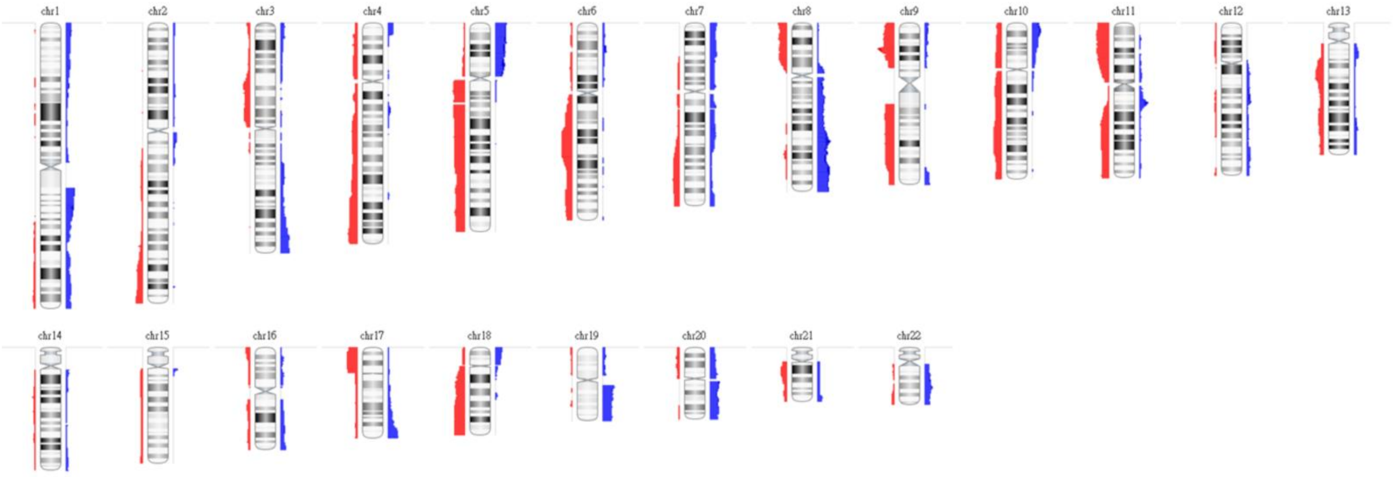

Figure-S1. Whole genome copy number imbalances of 40 UBUC samples focusing on somatic chromosomes. Copy number losses (red) are shown on the left and gains (blue) on the right of individual chromosomal ideograms generated with Nexus Copy Number™ software. Gains and losses on chromosomal regions are defined as the log2 ratios of corresponding probes signals being >=+0.20 or =<-0.20, respectively. The frequently altered somatic chromosome arms are +1q, -2q, -3p, +3q, -4q, +5p, -5q, -6q, +7p, -7q, -8p, -9q, +10p, -10q, -11p, +11q, -13q, -17p, +17q, +18p, -18q, +19q, +20, +22q.

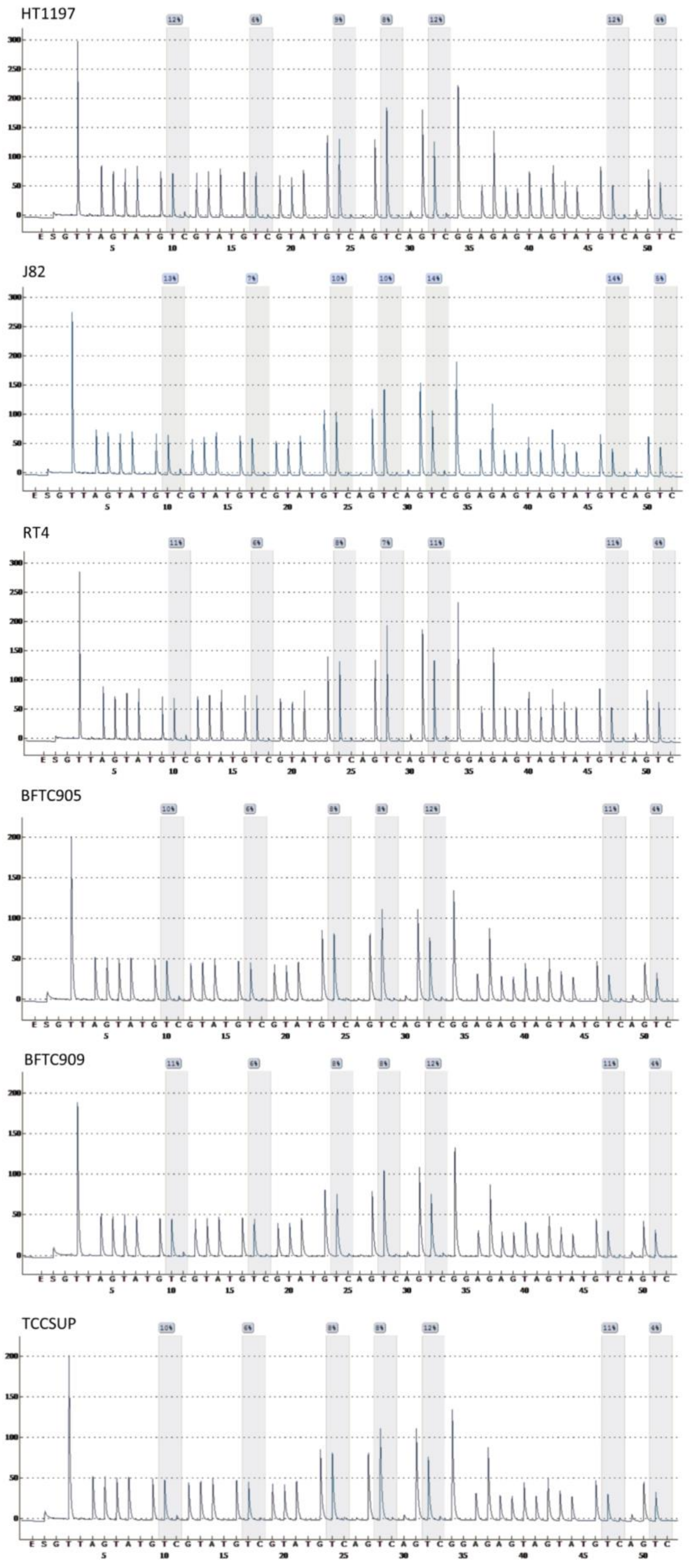

Figure-S2. Quantitative pyrosequencing for *CEBPD* promoter methylation status in UC cells. All cell lines including HT1197, J82, RT4, BFTC905, BTFC909, and TCCSUP show a very low degree (no more than 15%) of methylation in all CpG islands tested.

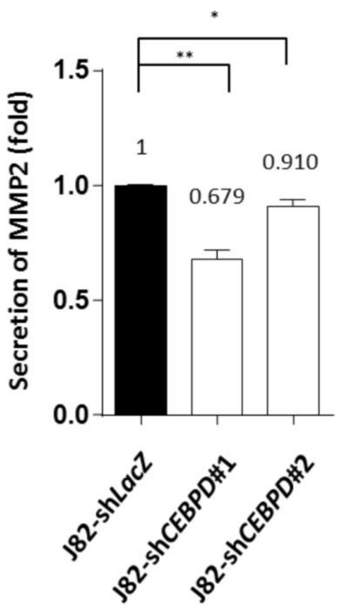

Figure S3. Measurement of MMP2 protein level in culture supernatant samples from J82 cells. Compared with control cells, CEBPD silencing significantly deplete secreted MMP2 levels in J82 cells.

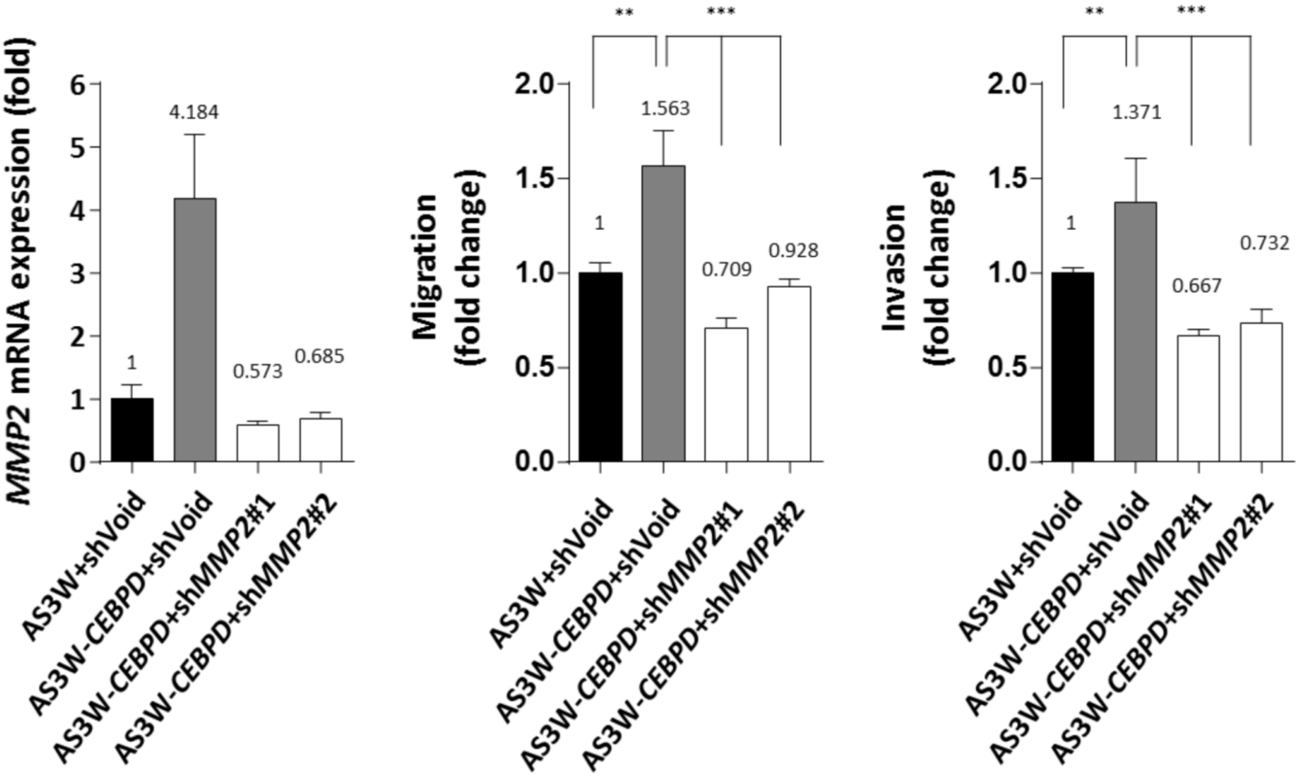

Figure S4. MMP2 silencing deplete CEBPD-driven cell migration and invasion in TCCSUP cells. After validation of *MMP2* Knockdown efficiency by quantitative RT-PCR (*left panel*), cell migration (*middle panel*) and invasion (*right panel*) assay was conducted which suggested MMP2 silencing significantly deplete exogenous CEBPD-induced cell migration and invasiveness in TCCSUP cells, confirming the role of MMP2 in CEBPD-driven aggressiveness.

Table-S1. Clinical pathological features of 40 UBUC samples submitted to aCGH

Tissue Banking No.	Gender	Age	pT status	pN status	Histological grade	Subsequent distal metastasis	Status
B1	M	83	T4	N1	High	Present	DOD
B13	M	71	T3	N0	High	Present	DUK
B3	F	78	T2	N0	High	Present	AWD
B16	M	91	T2	N0	High	Not identified	NED
B17	M	55	T2	N0	High	Not identified	NED
B5	F	77	T2	N0	High	Not identified	NED
B11	F	55	T1	N0	High	Not identified	NED
B7	F	68	T1	N0	High	Not identified	NED
CM1757	F	65	T1	N0	High	Not identified	NED
CM1760	F	66	T1	N0	High	Not identified	NED
CM1384	M	81	T1	N0	High	Present	DOD
CM1558	M	83	Ta	N0	High	Not identified	DUK
CM1746	F	81	Ta	N0	Low	Not identified	NED
CM1748	M	72	Ta	N0	High	Not identified	NED
CM1789	M	25	Ta	N0	Low	Not identified	NED
CM1354	M	65	T2	N0	High	Not identified	NED
CM1241	M	77	T2	N0	High	Not identified	NED
CM1685	M	65	T1	N0	High	Not identified	NED
25211427	M	85	T2	N0	High	Not identified	DUK
CM1679	M	75	T4	N0	High	Not identified	DUK
20510705	F	69	T2	N0	High	Present	DOD
CM1435	M	49	T1	N0	High	Not identified	NED
CM1438	M	64	T3a	N0	High	Not identified	NED
CM1476	F	71	T2	N0	High	Not identified	DUK
CM1484	M	55	T2	N2	High	Present	AWD
CM1497	M	81	T3	N0	High	Present	DOD

CM1526	M	76	T3	N0	High	Present	DOD
CM1469	M	85	T3	N2	High	Present	DOD
CM1552	M	81	T3	N2	High	Not identified	DUK
CM1578	M	52	T3	N0	High	Present	DOD
CM1670	F	77	T3	N0	High	Present	DOD
CM1526	M	76	T3	N0	High	Present	DOD
CM1594	M	87	T3	N0	High	Not identified	NED
CM1653	M	73	T3	N0	High	Not identified	NED
CM1679	M	75	T4	N0	High	Present	DOD
CM1681	M	57	T4	N1	High	Present	DOD
CM1688	F	75	T4	N0	High	Not identified	NED
CM1691	M	79	T4	N0	High	Present	DOD
CM1707	M	73	T4	N0	High	Present	DOD
CM1711	F	77	T4	N0	High	Not identified	DUK

Table-S2. Recurrent copy number aberrations identified by aCGH in 40 urothelial carcinomas of urinary bladder

Region	Region length	Cytoband Location	Event	Genes
chr1:142,756,696-168,092,275	25335579	q21.1 - q24.2	CN Gain	486
chr2:236,733,744-242,951,149	6217405	q37.2 - q37.3	CN Loss	63
chr3:52,557,272-57,694,373	5137101	p21.1 - p14.3	CN Loss	41
chr3:60,374,625-60,525,422	150797	p14.2	CN Loss	1
chr3:177,528,032-199,501,827	21973795	q26.32 - q29	CN Gain	158
chr4:150,345,165-191,273,063	40927898	q31.23 - q35.2	CN Loss	168
chr5:0-46,419,092	46419092	p15.33 - p11	CN Gain	149
chr5:49,596,616-68,756,175	19159559	q11.1 - q13.2	CN Loss	75
chr5:70,739,833-180,857,866	110118033	q13.2 - q35.3	CN Loss	664
chr6:77,485,696-124,847,059	47361363	q14.1 - q22.31	CN Loss	179
chr6:144,206,062-158,281,877	14075815	q24.2 - q25.3	CN Loss	62
chr6:162,890,981-163,191,639	300658	q26	CN Loss	2
chr7:0-2,982,212	2982212	p22.3 - p22.2	CN Gain	35
chr7:25,167,592-29,035,387	3867795	p15.2 - p15.1	CN Gain	30
chr7:134,775,923-136,888,688	2112765	q33	CN Loss	10
chr8:0-5,453,252	5453252	p23.3 - p23.2	CN Loss	12
chr8:5,771,237-6,048,216	276979	p23.2	CN Loss	0
chr8:6,146,543-34,279,185	28132642	p23.2 - p12	CN Loss	211
chr8:40,337,488-40,505,630	168142	p11.21	CN Gain	0
chr8:41,900,567-43,820,269	1919702	p11.21 - p11.1	CN Gain	18
chr8:48,553,626-49,593,636	1040010	q11.21	CN Gain	5
chr8:62,688,044-70,160,284	7472240	q12.3 - q13.2	CN Gain	33
chr8:74,554,643-75,482,882	928239	q21.11	CN Gain	7
chr8:80,877,379-81,149,978	272599	q21.13	CN Gain	2
chr8:85,622,907-85,902,318	279411	q21.2	CN Gain	1
chr8:86,526,515-87,877,141	1350626	q21.2 - q21.3	CN Gain	13

chr8:90,138,489-146,274,826	56136337	q21.3 - q24.3	CN Gain	283
chr9:0-32,725,713	32725713	p24.3 - p21.1	CN Loss	116
chr9:70,249,191-127,760,659	57511468	q13 - q33.3	CN Loss	343
chr10:0-1,882,485	1882485	p15.3	CN Gain	13
chr10:2,200,895-12,452,295	10251400	p15.3 - p13	CN Gain	48
chr10:54,593,311-63,881,731	9288420	q21.1 - q21.2	CN Loss	21
chr10:84,871,321-115,516,660	30645339	q23.1 - q25.3	CN Loss	244
chr11:0-47,239,358	47239358	p15.5 - p11.2	CN Loss	426
chr11:67,823,794-71,548,986	3725192	q13.2 - q13.4	CN Gain	37
chr11:81,290,093-134,452,384	53162291	q14.1 - q25	CN Loss	361
chr13:37,752,852-50,386,542	12633690	q13.3 - q14.3	CN Loss	80
chr17:0-22,200,000	22200000	p13.3 - q11.1	CN Loss	352
chr17:68,184,276-78,774,742	10590466	q24.3 - q25.3	CN Gain	206
chr18:0-4,137,612	4137612	p11.32 - p11.31	CN Gain	23
chr18:21,157,080-76,117,153	54960073	q11.2 - q23	CN Loss	191
chr19:32,651,846-63,811,651	31159805	q12 - q13.43	CN Gain	930
chr20:21,952,988-24,034,700	2081712	p11.22 - p11.21	CN Gain	21
chr20:29,309,964-62,435,964	33126000	q11.21 - q13.33	CN Gain	382
chr22:32,922,820-36,754,192	3831372	q12.3 - q13.1	CN Gain	53
chrX:0-467,573	467573	p22.33	CN Loss	3
chrX:0-2,700,568	2700568	p22.33	CN Gain	18
chrX:2,618,374-58,075,602	55457228	p22.33 - p11.1	CN Loss	405
chrX:62,794,454-154,913,754	92119300	q11.1 - q28	CN Loss	604

Table-S3. Comparing the whole genome copy number gains and losses between 13 DOD cases and the rest 27 cases

Region	Cytoband Location	Event	Genes	Region Length	Freq. in DOD case (%)	Freq. in non-DOD cases (%)	Difference (%)	p-value
chr8:47,043,376-48,553,626	q11.1 - q11.21	CN Gain	2	1510250	46.15385	3.703704	42.45014	0.0025771902
**chr8:48,553,626-49,593,636**	**q11.21**	**CN Gain**	**5**	**1040010**	**53.84615**	**3.703704**	**50.14245**	**0.0006191950**
chr8:49,593,636-50,240,262	q11.21	CN Gain	3	646626	46.15385	3.703704	42.45014	0.0025771902
chr8:81,149,978-85,622,907	q21.13 - q21.2	CN Gain	15	4472929	46.15385	3.703704	42.45014	0.0025771902
chr8:90,270,050-90,673,143	q21.3	CN Gain	0	403093	53.84615	7.407407	46.43875	0.0023324408
chr8:113,489,839-114,036,160	q23.3	CN Gain	1	546321	61.53846	11.11111	50.42735	0.0017403052
chr8:123,196,120-124,225,627	q24.13	CN Gain	3	1029507	69.23077	14.81481	54.41595	0.0011146249
chr8:80,877,379-81,149,978	q21.13	CN Gain	2	272599	46.15385	7.407407	38.74644	0.0084511756
chr8:85,622,907-85,902,318	q21.2	CN Gain	1	279411	46.15385	7.407407	38.74644	0.0084511756
chr8:86,526,515-87,877,141	q21.2 - q21.3	CN Gain	13	1350626	46.15385	7.407407	38.74644	0.0084511756
chr8:90,138,489-90,270,050	q21.3	CN Gain	0	131561	46.15385	7.407407	38.74644	0.0084511756
chr8:90,673,143-92,699,491	q21.3	CN Gain	11	2026348	46.15385	7.407407	38.74644	0.0084511756
chr8:110,149,187-110,317,937	q23.1	CN Gain	1	168750	53.84615	11.11111	42.73504	0.0064773902
chr8:111,705,076-113,253,734	q23.2 - q23.3	CN Gain	0	1548658	61.53846	14.81481	46.72365	0.0075471086
chr8:113,393,981-113,489,839	q23.3	CN Gain	1	95858	61.53846	14.81481	46.72365	0.0075471086
chr8:114,036,160-120,356,973	q23.3 - q24.12	CN Gain	13	6320813	53.84615	11.11111	42.73504	0.0064773902
chr8:122,013,540-123,196,120	q24.12 - q24.13	CN Gain	2	1182580	61.53846	14.81481	46.72365	0.0075471086
chr8:124,225,627-131,707,712	q24.13 - q24.22	CN Gain	33	7482085	69.23077	18.51852	50.71225	0.0035779093
chr8:133,446,731-133,738,793	q24.22	CN Gain	3	292062	61.53846	14.81481	46.72365	0.0075471086
chr8:137,726,857-142,019,273	q24.23 - q24.3	CN Gain	7	4292416	61.53846	14.81481	46.72365	0.0075471086
chr8:50,240,262-50,256,881	q11.21	CN Gain	0	16619	38.46154	3.703704	34.75783	0.0095001016
chr8:131,707,712-133,446,731	q24.22	CN Gain	5	1739019	61.53846	18.51852	43.01994	0.0114159225
chr8:133,738,793-137,726,857	q24.22 - q24.23	CN Gain	12	3988064	61.53846	18.51852	43.01994	0.0114159225
chr8:142,019,273-146,274,826	q24.3	CN Gain	105	4255553	61.53846	18.51852	43.01994	0.0114159225
chr9:8,345,416-8,689,698	p24.1	CN Loss	1	344282	0	44.44444	-44.4444	0.0035042516
chr9:9,260,539-9,688,589	p23	CN Loss	1	428050	0	44.44444	-44.4444	0.0035042516

chr9:11,713,128-13,903,388	p23	CN Loss	3	2190260	0	44.44444	-44.4444	0.0035042516
chr9:13,903,388-14,254,369	p23 - p22.3	CN Loss	1	350981	0	48.14815	-48.1481	0.0027815349
chr9:14,254,369-20,872,862	p22.3 - p21.3	CN Loss	24	6618493	0	44.44444	-44.4444	0.0035042516
chr3:88,296,222-90,346,746	p11.2 - p11.1	CN Loss	1	2050524	30.76923	0	30.76923	0.0078236131
chr9:2,288,487-3,230,947	p24.2	CN Loss	5	942460	0	40.74074	-40.7407	0.0073800175
chr9:3,348,758-4,566,309	p24.2	CN Loss	4	1217551	0	40.74074	-40.7407	0.0073800175
chr9:5,602,044-8,345,416	p24.1	CN Loss	12	2743372	0	40.74074	-40.7407	0.0073800175
chr9:8,689,698-9,260,539	p24.1 - p23	CN Loss	1	570841	0	40.74074	-40.7407	0.0073800175
chr9:10,244,410-11,713,128	p23	CN Loss	1	1468718	0	40.74074	-40.7407	0.0073800175
chr5:29,828,158-30,489,025	p13.3	CN Gain	0	660867	0	33.33333	-33.3333	0.0194727429
chr5:41,296,835-44,507,527	p13.1 - p12	CN Gain	19	3210692	0	33.33333	-33.3333	0.0194727429
chr8:108,077,048-108,233,083	q23.1	CN Gain	0	156035	53.84615	14.81481	39.03134	0.0204070013
chr8:110,317,937-111,705,076	q23.1 - q23.2	CN Gain	6	1387139	53.84615	14.81481	39.03134	0.0204070013
chr8:113,253,734-113,393,981	q23.3	CN Gain	1	140247	53.84615	14.81481	39.03134	0.0204070013
chr8:120,356,973-122,013,540	q24.12	CN Gain	9	1656567	53.84615	14.81481	39.03134	0.0204070013
chr8:70,160,284-74,554,643	q13.2 - q21.11	CN Gain	15	4394359	38.46154	7.407407	31.05413	0.0268073801
chr8:75,482,882-78,288,332	q21.11	CN Gain	6	2805450	38.46154	7.407407	31.05413	0.0268073801
chr8:79,807,898-80,877,379	q21.12 - q21.13	CN Gain	3	1069481	38.46154	7.407407	31.05413	0.0268073801
chr8:85,902,318-86,526,515	q21.2	CN Gain	6	624197	38.46154	7.407407	31.05413	0.0268073801
chr8:87,877,141-90,138,489	q21.3	CN Gain	3	2261348	38.46154	7.407407	31.05413	0.0268073801
chr9:1,110,590-1,448,581	p24.3	CN Loss	0	337991	0	37.03704	-37.037	0.0164298237
chr9:1,714,125-1,822,850	p24.3	CN Loss	0	108725	0	37.03704	-37.037	0.0164298237
chr9:1,890,727-2,288,487	p24.3 - p24.2	CN Loss	1	397760	0	37.03704	-37.037	0.0164298237
chr9:3,230,947-3,348,758	p24.2	CN Loss	1	117811	0	37.03704	-37.037	0.0164298237
chr9:4,566,309-5,602,044	p24.2 - p24.1	CN Loss	14	1035735	0	37.03704	-37.037	0.0164298237
chr9:21,140,218-21,359,345	p21.3	CN Loss	11	219127	7.692308	48.14815	-40.4558	0.0148141177
chr9:0-1,110,590	p24.3	CN Loss	12	1110590	0	33.33333	-33.3333	0.0194727429
chr9:1,448,581-1,714,125	p24.3	CN Loss	0	265544	0	33.33333	-33.3333	0.0194727429
chr9:1,822,850-1,890,727	p24.3	CN Loss	0	67877	0	33.33333	-33.3333	0.0194727429
chr8:50,256,881-51,471,323	q11.21 - q11.22	CN Gain	1	1214442	30.76923	3.703704	27.06553	0.0312944523

chr8:107,977,791-108,077,048	q23.1	CN Gain	0	99257	53.84615	18.51852	35.32764	0.0323432985
chr5:30,489,025-30,866,383	p13.3	CN Gain	0	377358	0	29.62963	-29.6296	0.0373190528
chr5:44,507,527-46,419,092	p12 - p11	CN Gain	2	1911565	0	29.62963	-29.6296	0.0373190528
chr8:92,699,491-93,529,070	q21.3 - q22.1	CN Gain	1	829579	46.15385	11.11111	35.04274	0.0378289474
chr8:109,782,370-110,149,187	q23.1	CN Gain	1	366817	46.15385	11.11111	35.04274	0.0378289474
chr3:88,030,008-88,296,222	p11.2	CN Loss	4	266214	30.76923	3.703704	27.06553	0.0312944523
chr9:9,688,589-9,757,038	p23	CN Loss	1	68449	7.692308	44.44444	-36.7521	0.0301964423
chr9:20,872,862-21,140,218	p21.3	CN Loss	4	267356	7.692308	44.44444	-36.7521	0.0301964423
chr11:43,891,647-47,239,358	p11.2	CN Loss	40	3347711	0	29.62963	-29.6296	0.0373190528

Table-S4. Comparing the whole genome copy number gains and losses between 16 cases developed distal metastasis and the rest 24.

Region	Cytoband Location	Event	Genes	Region Length	Freq. in those with Meta. (%)	Freq. in Non-Meta (%)	Difference (%)	p-value
chr8:123,196,120-124,225,627	q24.13	CN Gain	3	1029507	75	4.166666667	70.83333	0.0000036765
chr8:122,013,540-123,196,120	q24.12 - q24.13	CN Gain	2	1182580	68.75	4.166666667	64.58333	0.0000190898
chr8:124,225,627-131,707,712	q24.13 - q24.22	CN Gain	33	7482085	75	8.333333333	66.66667	0.0000222296
chr8:113,489,839-114,036,160	q23.3	CN Gain	1	546321	62.5	4.166666667	58.33333	0.0000850246
chr8:120,356,973-122,013,540	q24.12	CN Gain	9	1656567	62.5	4.166666667	58.33333	0.0000850246
chr8:131,707,712-133,446,731	q24.22	CN Gain	5	1739019	68.75	8.333333333	60.41667	0.0001038631
**chr8:48,553,626-49,593,636**	**q11.21**	**CN Gain**	**5**	**1040010**	**50**	**0**	**50**	**0.0001673500**
chr8:107,977,791-108,077,048	q23.1	CN Gain	0	99257	62.5	8.333333333	54.16667	0.0004146985
chr8:110,149,187-110,317,937	q23.1	CN Gain	1	168750	56.25	4.166666667	52.08333	0.0003333504
chr8:111,705,076-113,253,734	q23.2 - q23.3	CN Gain	0	1548658	62.5	8.333333333	54.16667	0.0004146985
chr8:113,393,981-113,489,839	q23.3	CN Gain	1	95858	62.5	8.333333333	54.16667	0.0004146985
chr8:114,036,160-120,356,973	q23.3 - q24.12	CN Gain	13	6320813	56.25	4.166666667	52.08333	0.0003333504
chr8:133,446,731-133,738,793	q24.22	CN Gain	3	292062	62.5	8.333333333	54.16667	0.0004146985
chr8:47,043,376-48,553,626	q11.1 - q11.21	CN Gain	2	1510250	43.75	0	43.75	0.0006136167
chr8:49,593,636-50,240,262	q11.21	CN Gain	3	646626	43.75	0	43.75	0.0006136167
chr8:81,149,978-85,622,907	q21.13 - q21.2	CN Gain	15	4472929	43.75	0	43.75	0.0006136167
chr8:90,270,050-90,673,143	q21.3	CN Gain	0	403093	50	4.166666667	45.83333	0.0011714501
chr8:109,782,370-110,149,187	q23.1	CN Gain	1	366817	50	4.166666667	45.83333	0.0011714501
chr8:133,738,793-137,726,857	q24.22 - q24.23	CN Gain	12	3988064	62.5	12.5	50	0.0016582543
chr8:50,240,262-50,256,881	q11.21	CN Gain	0	16619	37.5	0	37.5	0.0020862968
chr8:105,405,416-105,866,539	q22.3	CN Gain	3	461123	56.25	8.333333333	47.91667	0.0025305564
chr8:106,746,387-107,977,791	q23.1	CN Gain	3	1231404	56.25	8.333333333	47.91667	0.0025305564
chr8:108,077,048-108,233,083	q23.1	CN Gain	0	156035	56.25	8.333333333	47.91667	0.0025305564
chr8:110,317,937-111,705,076	q23.1 - q23.2	CN Gain	6	1387139	56.25	8.333333333	47.91667	0.0025305564
chr8:113,253,734-113,393,981	q23.3	CN Gain	1	140247	56.25	8.333333333	47.91667	0.0025305564

chr8:65,293,366-70,160,284	q12.3 - q13.2	CN Gain	27	4866918	43.75	4.166666667	39.58333	0.0037374836
chr8:80,877,379-81,149,978	q21.13	CN Gain	2	272599	43.75	4.166666667	39.58333	0.0037374836
chr8:85,622,907-85,902,318	q21.2	CN Gain	1	279411	43.75	4.166666667	39.58333	0.0037374836
chr8:86,526,515-87,877,141	q21.2 - q21.3	CN Gain	13	1350626	43.75	4.166666667	39.58333	0.0037374836
chr8:90,138,489-90,270,050	q21.3	CN Gain	0	131561	43.75	4.166666667	39.58333	0.0037374836
chr8:90,673,143-92,699,491	q21.3	CN Gain	11	2026348	43.75	4.166666667	39.58333	0.0037374836
chr9:2,288,487-3,230,947	p24.2	CN Loss	5	942460	0	45.83333333	-45.8333	0.0011647644
chr9:3,348,758-4,566,309	p24.2	CN Loss	4	1217551	0	45.83333333	-45.8333	0.0011647644
chr9:5,602,044-8,345,416	p24.1	CN Loss	12	2743372	0	45.83333333	-45.8333	0.0011647644
chr9:8,345,416-8,689,698	p24.1	CN Loss	1	344282	0	50	-50	0.0008987198
chr9:8,689,698-9,260,539	p24.1 - p23	CN Loss	1	570841	0	45.83333333	-45.8333	0.0011647644
chr9:9,260,539-9,688,589	p23	CN Loss	1	428050	0	50	-50	0.0008987198
chr9:10,244,410-11,713,128	p23	CN Loss	1	1468718	0	45.83333333	-45.8333	0.0011647644
chr8:98,183,770-99,509,907	q22.1 - q22.2	CN Gain	12	1326137	56.25	12.5	43.75	0.0050431922
chr8:105,248,164-105,405,416	q22.3	CN Gain	1	157252	56.25	12.5	43.75	0.0050431922
chr8:105,866,539-106,746,387	q22.3 - q23.1	CN Gain	1	879848	56.25	12.5	43.75	0.0050431922
chr8:137,726,857-142,019,273	q24.23 - q24.3	CN Gain	7	4292416	56.25	12.5	43.75	0.0050431922
chr5:41,296,835-44,507,527	p13.1 - p12	CN Gain	19	3210692	0	37.5	-37.5	0.0059531549
chr8:50,256,881-51,471,323	q11.21 - q11.22	CN Gain	1	1214442	31.25	0	31.25	0.0066382172
chr8:108,233,083-109,782,370	q23.1	CN Gain	4	1549287	50	8.333333333	41.66667	0.0068375768
chr9:1,110,590-1,448,581	p24.3	CN Loss	0	337991	0	41.66666667	-41.6667	0.0026470785
chr9:1,714,125-1,822,850	p24.3	CN Loss	0	108725	0	41.66666667	-41.6667	0.0026470785
chr9:1,890,727-2,288,487	p24.3 - p24.2	CN Loss	1	397760	0	41.66666667	-41.6667	0.0026470785
chr9:3,230,947-3,348,758	p24.2	CN Loss	1	117811	0	41.66666667	-41.6667	0.0026470785
chr9:4,566,309-5,602,044	p24.2 - p24.1	CN Loss	14	1035735	0	41.66666667	-41.6667	0.0026470785
chr8:54,725,239-57,920,248	q11.23 - q12.1	CN Gain	20	3195009	37.5	4.166666667	33.33333	0.0109223775
chr8:70,160,284-74,554,643	q13.2 - q21.11	CN Gain	15	4394359	37.5	4.166666667	33.33333	0.0109223775
chr8:75,482,882-78,288,332	q21.11	CN Gain	6	2805450	37.5	4.166666667	33.33333	0.0109223775

chr8:79,807,898-80,877,379	q21.12 - q21.13	CN Gain	3	1069481	37.5	4.166666667	33.33333	0.0109223775
chr8:85,902,318-86,526,515	q21.2	CN Gain	6	624197	37.5	4.166666667	33.33333	0.0109223775
chr8:87,877,141-90,138,489	q21.3	CN Gain	3	2261348	37.5	4.166666667	33.33333	0.0109223775
chr9:0-1,110,590	p24.3	CN Loss	12	1110590	0	37.5	-37.5	0.0059531549
chr9:1,448,581-1,714,125	p24.3	CN Loss	0	265544	0	37.5	-37.5	0.0059531549
chr9:1,822,850-1,890,727	p24.3	CN Loss	0	67877	0	37.5	-37.5	0.0059531549
chr9:9,688,589-9,757,038	p23	CN Loss	1	68449	6.25	50	-43.75	0.0052538410
chr9:13,903,388-14,254,369	p23 - p22.3	CN Loss	1	350981	6.25	50	-43.75	0.0052538410
chr5:30,489,025-30,866,383	p13.3	CN Gain	0	377358	0	33.33333333	-33.3333	0.0133008932
chr5:44,507,527-46,419,092	p12 - p11	CN Gain	2	1911565	0	33.33333333	-33.3333	0.0133008932
chr8:95,087,177-98,183,770	q22.1	CN Gain	18	3096593	50	12.5	37.5	0.0137983407
chr8:99,509,907-99,574,803	q22.2	CN Gain	2	64896	56.25	16.66666667	39.58333	0.0153559925
chr8:104,286,371-105,248,164	q22.3	CN Gain	6	961793	56.25	16.66666667	39.58333	0.0153559925
chr8:142,019,273-146,274,826	q24.3	CN Gain	105	4255553	56.25	16.66666667	39.58333	0.0153559925
chr8:63,930,325-65,293,366	q12.3	CN Gain	5	1363041	43.75	8.333333333	35.41667	0.0175003057
chr8:92,699,491-93,529,070	q21.3 - q22.1	CN Gain	1	829579	43.75	8.333333333	35.41667	0.0175003057
chr9:9,757,038-10,244,410	p23	CN Loss	1	487372	6.25	45.83333333	-39.5833	0.0121918143
chr9:11,713,128-13,903,388	p23	CN Loss	3	2190260	6.25	45.83333333	-39.5833	0.0121918143
chr9:14,254,369-20,872,862	p22.3 - p21.3	CN Loss	24	6618493	6.25	45.83333333	-39.5833	0.0121918143
chr11:43,891,647-47,239,358	p11.2	CN Loss	40	3347711	0	33.33333333	-33.3333	0.0133008932
chr8:51,471,323-54,725,239	q11.22 - q11.23	CN Gain	8	3253916	31.25	4.166666667	27.08333	0.0293978189
chr8:57,920,248-62,467,446	q12.1 - q12.3	CN Gain	12	4547198	31.25	4.166666667	27.08333	0.0293978189
chr8:78,288,332-79,807,898	q21.11 - q21.12	CN Gain	3	1519566	31.25	4.166666667	27.08333	0.0293978189
chr5:31,085,310-34,670,174	p13.3 - p13.2	CN Gain	16	3584864	6.25	37.5	-31.25	0.0315173435
chr5:38,037,083-41,296,835	p13.2 - p13.1	CN Gain	16	3259752	6.25	37.5	-31.25	0.0315173435
chr8:99,574,803-99,724,951	q22.2	CN Gain	1	150148	50	16.66666667	33.33333	0.0366701043
chr3:88,296,222-90,346,746	p11.2 - p11.1	CN Loss	1	2050524	25	0	25	0.0199146515
chr5:147,856,171-161,321,465	q33.1 - q34	CN Loss	96	13465294	6.25	37.5	-31.25	0.0315173435

chr7:100,903,295-107,401,392	q22.1 - q31.1	CN Loss	54	6498097	25	0	25	0.0199146515
chr7:112,383,354-116,739,942	q31.1 - q31.2	CN Loss	17	4356588	31.25	4.166666667	27.08333	0.0293978189
chr9:21,140,218-21,359,345	p21.3	CN Loss	11	219127	12.5	50	-37.5	0.0198582063
chr9:29,133,938-31,362,826	p21.1	CN Loss	0	2228888	6.25	37.5	-31.25	0.0315173435
chr9:32,725,713-38,358,845	p21.1 - p13.1	CN Loss	97	5633132	0	29.16666667	-29.1667	0.0294866431
chr9:70,249,191-78,298,983	q13 - q21.13	CN Loss	35	8049792	6.25	37.5	-31.25	0.0315173435
chr9:78,298,983-84,180,437	q21.13 - q21.32	CN Loss	17	5881454	6.25	41.66666667	-35.4167	0.0273722124
chr9:84,180,437-95,746,200	q21.32 - q22.32	CN Loss	66	11565763	6.25	37.5	-31.25	0.0315173435
chr9:95,746,200-96,861,944	q22.32	CN Loss	8	1115744	6.25	41.66666667	-35.4167	0.0273722124
chr9:96,861,944-104,049,320	q22.32 - q31.1	CN Loss	59	7187376	6.25	37.5	-31.25	0.0315173435
chr9:104,049,320-104,743,068	q31.1	CN Loss	0	693748	6.25	41.66666667	-35.4167	0.0273722124
chr9:104,743,068-123,090,251	q31.1 - q33.2	CN Loss	106	18347183	6.25	37.5	-31.25	0.0315173435
chr9:127,760,659-128,750,793	q33.3	CN Loss	6	990134	0	29.16666667	-29.1667	0.0294866431
chr11:37,640,112-38,452,907	p12	CN Loss	0	812795	6.25	37.5	-31.25	0.0315173435
chr11:38,452,907-39,244,329	p12	CN Loss	0	791422	6.25	41.66666667	-35.4167	0.0273722124
chr11:39,244,329-40,781,670	p12	CN Loss	1	1537341	6.25	37.5	-31.25	0.0315173435
chr11:47,239,358-47,588,438	p11.2	CN Loss	12	349080	0	29.16666667	-29.1667	0.0294866431
chr14:42,721,124-42,791,031	q21.2	CN Loss	0	69907	25	0	25	0.0199146515
chr3:177,528,032-183,063,140	q26.32 - q26.33	CN Gain	19	5535108	37.5	8.333333333	29.16667	0.0420404687
chr8:41,900,567-43,820,269	p11.21 - p11.1	CN Gain	18	1919702	37.5	8.333333333	29.16667	0.0420404687
chr8:62,688,044-63,930,325	q12.3	CN Gain	2	1242281	37.5	8.333333333	29.16667	0.0420404687
chr8:74,554,643-75,482,882	q21.11	CN Gain	7	928239	37.5	8.333333333	29.16667	0.0420404687
chr8:103,172,939-104,286,371	q22.3	CN Gain	9	1113432	56.25	20.83333333	35.41667	0.0408108167
chr22:35,577,810-36,754,192	q12.3 - q13.1	CN Gain	32	1176382	37.5	8.333333333	29.16667	0.0420404687

Table-S5. CEBPD gene dosage, promoter methylation, and protein expression statuses in selected UBUCs

Sample ID	*CEBPD* gene status	*CEBPD* promoter CpG methylation (%)*	CEBPD Expression#
U126	**Amplification**	**Methylated (32.184)**	Low
U135	Non-amplified	Unmethylated (18.805)	**High**
U175	Non-amplified	Unmethylated (10.859)	Low
U176	**Amplification**	Unmethylated (15.71)	**High**
U181	Non-amplified	Unmethylated (2.724)	Low
U182	**Amplification**	Unmethylated (11.21)	**High**
U188	Non-amplified	Unmethylated (3.794)	Low
U190	Non-amplified	**Methylated (20.013)**	Low
U198	Non-amplified	Unmethylated (17.178)	Low
U217	**Amplification**	Unmethylated (12.082)	**High**
U220	Non-amplified	Unmethylated (10.896)	Low
U223	**Amplification**	Unmethylated (8.754)	**High**
U228	Non-amplified	Unmethylated (14.195)	Low
U235	Non-amplified	Unmethylated (2.294)	Low
U236	Non-amplified	Unmethylated (7.12)	**High**
U242	Non-amplified	Unmethylated (11.213)	**High**
U248	Non-amplified	Unmethylated (12.587)	Low
U253	Non-amplified	Unmethylated (10.275)	Low
U254	Non-amplified	Unmethylated (2.324)	Low
U262	Non-amplified	Unmethylated (11.809)	Low

*,The cut-off values used to define methylation is 19.324%, based on the mean+3 s.d. of the percentages of CpG methylation from normal epithelia.

#,Computerized by Chi-square analysis, CEBPD protein expression is significantly associated with gene amplification (*P*=0.031) but not promoter methylation (*P*=0.512).

Table-S6. Correlations between *CEBPD* amplification and protein expression and other important clinicopathological parameters in urothelial carcinoma of upper urinary tract.

Parameter	Category	Case No.	*CEBPD* Gene Amplification			CEBPD Expression		
			Non-Amp.	Amp.	p-value	Low	High	p-value
Gender[&]	Male	158	123	35	0.934	114	44	0.514
	Female	182	141	41		137	45	
Age (years)[#]		340	65.5+/-10.2	65.9+/-8.7	0.889	65.6+/-10.0	65.6+/-9.7	0.737
Tumor location[&]	Renal pelvis	141	109	32	0.124	103	38	0.420
	Ureter	150	122	28		115	35	
	Renal pelvis & ureter	49	33	16		33	16	
Multifocality[&]	Single	278	220	58	0.163	209	69	0.228
	Multifocal	62	44	18		42	20	
Primary tumor (T) [&]	Ta	89	81	8	**<0.001***	72	17	**<0.001***
	T1	92	76	16		78	14	
	T2-T4	159	107	52		101	58	
Nodal metastasis[&]	Negative (N0)	312	248	64	**0.007***	241	71	**<0.001***
	Positive (N1-N2)	28	16	12		10	18	
Histological grade [&]	Low grade	56	52	4	**0.003***	50	6	**0.004***
	High grade	284	212	72		201	83	
Vascular invasion[&]	Absent	234	197	37	**<0.001***	188	46	**<0.001***
	Present	106	67	39		63	43	
Perineural invasion[&]	Absent	321	254	67	**0.007***	243	78	**0.001***
	Present	19	10	9		8	11	
Mitotic rate (per 10 high power fields)[#]		340	11.9+/-12.3	13.6+/-12.2	0.064	11.8+/-12.5	13.7+/-11.6	**0.011***
CEBPD expression[&]	Low Expression	251	244	7	**<0.001***	-	-	-
	High Expression	89	20	69		-	-	-
MMP2 expression[&]	Low Expression	223	194	29	**<0.001***	191	32	**<0.001***
	High Expression	117	70	47		60	57	

&, Chi-Square test; #, Mann-Whitney U test; * Statistically significant

Table-S7. Univariate log-rank and multivariate analyses for Disease-specific and Metastasis-free Survivals in upper urinary tract urothelial carcinoma

**Parameter**	**Category**	Case No.	**Disease-specific Survival**					**Metastasis-free Survival**				
			Univariate analysis		Multivariate analysis			Univariate analysis		Multivariate analysis		
			No. of event	p-value	R.R.	95% C.I.	p-value	No. of event	p-value	R.R.	95% C.I.	p-value
**Gender**	Male	158	28	0.9301	-	-	-	32	0.7904	-	-	-
	Female	182	33		-	-	-	38		-	-	-
**Age (years)**	< 65	138	26	0.8660	-	-	-	30	0.8470	-	-	-
	≥ 65	202	35		-	-	-	40		-	-	-
**Tumor side**	Right	177	34	0.7188	-	-	-	38	0.3074	-	-	-
	Left	154	26		-	-	-	32		-	-	-
	Bilateral	9	1		-	-	-	0		-	-	-
**Tumor location**	Renal pelvis	141	24	**0.0120***	1			31	0.0659			
	Ureter	150	22					25				
	Renal pelvis & ureter	49	15					14				
**Multifocality**	Single	273	43	**0.0042***	1	-	**0.013**	52	**0.0196***	1	-	**0.002***
	Multifocal	62	18		2.680	1.227-5.854		18		1.942	1.273-2.964	
**Primary tumor (T)**	Ta	89	2	**<0.0001***	1	-	**0.035***	4	**<0.0001***	1	-	0.421
	T1	92	9		3.886	0.812-18.607		15		1.390	0.738-2.619	
	T2-T4	159	50		6.686	1.481-30.192		51		1.517	0.806-2.855	
**Nodal metastasis**	Negative (N0)	312	42	**<0.0001***	1	-	**<0.001***	55	**<0.0001***	1	-	**0.004***
	Positive (N1-N2)	28	19		3.866	1.957-7.637		15		2.225	1.288-3.844	
**Histological grade**	Low grade	56	4	**0.0171***	1	-	0.114	3	**0.0019***	1	-	0.165
	High grade	284	57		2.507	0.802-7.834		67		1.637	0.817-3.282	
**Vascular invasion**	Absent	234	24	**<0.0001***	1	-	0.100	26	**<0.0001***	1	-	**0.035***
	Present	106	37		1.662	0.908-3.041		44		1.655	1.037-2.643	
**Perineural invasion**	Absent	321	50	**<0.0001***	1	-	**0.006***	61	**<0.0001***	1	-	**0.007***
	Present	19	11		2.936	1.362-6.325		9		2.311	1.252-4.266	
**Mitotic rate (per 10 high power fields)**	< 10	173	27	0.1268	-	-	-	30	0.0581	-	-	-
	>= 10	167	34		-	-	-	40		-	-	-
**CEBPD Amplification**	Non-amplified	264	29	**<0.0001***	-	-	-	24	**<0.0001***	-	-	-
	Amplified	76	32		-	-	-	46		-	-	-
**CEBPD expression**	Low	251	26	**<0.0001***	1	-	**0.015***	24	**<0.0001***	1	-	**<0.001**
	High	89	35		2.168	1.161-4.047		46		2.648	1.709-4.104	
**MMP2 expression**	Low	223	27	**<0.0001**	1	-	0.892	36	**0.0030***	1	-	0.799
	High	117	34		1.044	0.562-1.938		34		0.946	0.620-1.444	

*** Statistically significant**

Table-S8. Cell cycle alteration of urothelial carcinoma cells with CEBPD manipulaiton

**Cell line-Conditions**	**G1 Phase** (Percent, Mean±SD)	***P*-Value**	**S Phase** (Percent, Mean±SD)	***P*-Value**	**G2/M Phase** (Percent, Mean±SD)	***P*-Value**
AS3W	71.02±1.85		20.23±2.43		6.80±1.49	
AS3W-*CEBPD*	68.23±1.54	$P < 0.05$	20.22±1.63	N.S.	11.23±1.26	$P < 0.01$
HT1197-sh*LacZ*	50.99±1.42		20.51±1.22		28.46±0.67	
HT1197-sh*CEBPD*#1	58.68±1.74	$P < 0.005$	11.67±1.31	$P < 0.005$	29.77±1.16	N.S.
HT1197-sh*CEBPD*#2	56.69±0.90	$P < 0.005$	17.08±0.58	$P < 0.005$	26.01±0.53	$P < 0.005$
J82-sh*LacZ*	52.07±1.26		29.46±1.94		18.11±1.52	
J82-sh*CEBPD*#1	64.86±2.01	$P < 0.005$	20.46±1.19	$P < 0.005$	15.24±0.73	N.S.
J82-sh*CEBPD*#2	63.49±0.93	$P < 0.005$	23.95±1.89	$P < 0.05$	13.25±0.30	$P < 0.005$

Abbreviation: N.S.:, non-significant

Table-S9. Differentially expressed tumor metastasis-associated genes selected from RT-PCR expression array

**Symbol**	**Refseq**	**Description**	TCCSUP CEBPD Overexpression		HT1197 CEBPD Knowndown		J82 CEBPD Knowndown	
			Fold Change	p-value	Fold Change	p-value	Fold Change	p-value
MMP13	NM_002427	Matrix metallopeptidase 13 (collagenase 3)	-3.986	<0.000001	-1.008	0.982493	-2.857	0.000356
MCAM	NM_006500	Melanoma cell adhesion molecule	-2.834	0.013302	2.069	<0.000001	1.416	0.059933
TIMP4	NM_003256	TIMP metallopeptidase inhibitor 4	2.005	<0.000001	2.007	<0.000001	-1.002	0.989578
CD44	NM_000610	CD44 molecule (Indian blood group)	1.995	<0.000001	2.002	<0.000001	-1.001	0.994376
TIMP3	NM_000362	TIMP metallopeptidase inhibitor 3	3.981	<0.000001	1.992	<0.000001	1.415	0.064569
MMP2	**NM_004530**	**Matrix metallopeptidase 2 (gelatinase A, 72kDa gelatinase, 72kDa type IV collagenase)**	**2.821**	**0.013858**	**-2.014**	**<0.000001**	**-1.43**	**0.047728**
NR4A3	NM_006981	Nuclear receptor subfamily 4, group A, member 3	1.957	<0.000001	-2.875	0.000128	1.411	0.06063
TNFSF10	NM_003810	Tumor necrosis factor (ligand) superfamily, member 10	2.007	<0.000001	-4.011	<0.000001	-1.002	0.989641

Table-S10. Correlations between MMP2 expression and other important clinicopathological parameters in urothelial carcinoma of urinary bladder and upper urinary tract.

Parameter	Category	Urinary Bladder Urothelial Carcinoma				Upper Urinary Tract Urothelial Carcinoma			
		Case No.	MMP2 Expression		p-value	Case No.	MMP2 Expression		p-value
			Low	High			Low	High	
Gender[&]	Male	216	136	80	0.413	158	107	51	0.493
	Female	79	54	25		182	116	66	
Age (years)[#]		295	65.9+/-12.4	66.3+/-11.9	0.953	340	65.2+/-10.0	65.4+/-9.8	0.341
Tumor location[&]	Renal pelvis	-	-	-	-	141	94	47	0.937
	Ureter	-	-	-	-	150	97	53	
	Renal pelvis & ureter	-	-	-	-	49	32	17	
Multifocality[&]	Single	-	-	-	-	278	181	97	0.768
	Multifocal	-	-	-	-	62	42	20	
Primary tumor (T) [&]	Ta	84	66	18	**<0.001***	89	65	24	**0.002***
	T1	88	66	22		92	69	23	
	T2-T4	123	58	65		159	89	70	
Nodal metastasis[&]	Negative (N0)	266	181	85	**<0.001***	312	215	97	**<0.001***
	Positive (N1-N2)	29	9	20		28	8	20	
Histological grade [&]	Low grade	56	41	15	0.163	56	40	16	0.314
	High grade	239	149	90		284	183	101	
Vascular invasion[&]	Absent	275	166	80	**0.021***	234	167	67	**0.001***
	Present	20	24	25		106	56	50	
Perineural invasion[&]	Absent	275	181	94	0.088	321	216	105	**0.007***
	Present	20	9	11		19	7	12	
Mitotic rate (per 10 high power fields)[#]		295	12.8+/-13.9	17.4+/-13.8	**0.001***	340	11.3+/-12.0	14.2+/-12.7	**0.013***

&, Chi-Square test; #, Mann-Whitney U test; * Statistically significant
